# Exploration of the In Vitro and In Vivo Neuroprotective Effects of Several Polyphenolics on LPS‐Induced Neuroinflammation

**DOI:** 10.1155/bri/1541212

**Published:** 2026-04-13

**Authors:** Mona Elkhatieb, Rasha A. Radwan, Doaa Abou El-ezz, Christine Adel Sedky, Ulrike Breitinger, Nabila Hamdi, Amira Emad Abdelaziz, Sarah Atef Fahim, Ahmed M. Hafez

**Affiliations:** ^1^ Department of Biochemistry, Faculty of Pharmacy and Biotechnology, German University in Cairo (GUC), Cairo, 11835, Egypt; ^2^ Biochemistry Department, Faculty of Biotechnology, German International University, Regional Ring Road East Cairo New Administrative Capital, Cairo, Egypt; ^3^ Department of Pharmacology and Toxicology, Faculty of Pharmacy, October University for Modern Sciences and Arts (MSA), Giza, 12556, Egypt, msa.edu.eg; ^4^ Department of Pharmacology and Toxicology, Faculty of Pharmacy and Biotechnology, German University in Cairo (GUC), Cairo, 11835, Egypt; ^5^ Pharmacology Department, Clinical and Biological Sciences Departments, College of Pharmacy, Arab Academy for Science and Technology and Maritime Transport, Abu Kir, Alexandria, Egypt, aast.edu; ^6^ Basic Sciences Department, Faculty of Physical Therapy, Egyptian Chinese University, Cairo, Egypt; ^7^ Department of Biochemistry, School of Life and Medical Sciences, University of Hertfordshire Hosted by Global Academic Foundation, Cairo, 11586, Egypt

**Keywords:** curcumin, hesperidin, isoliquiritigenin, neuroinflammation

## Abstract

Oxidative stress and neuroinflammation are key components in neurodegenerative diseases, where early intervention using natural treatments may offer neuroprotective effects. This study shows that isoliquiritigenin (ISL), hesperidin (HES), and curcumin (CUR) can mitigate lipopolysaccharide (LPS)‐induced neuroinflammation and oxidative stress in vitro and in vivo. The compounds were initially tested for cytotoxicity and found to reduce nitric oxide (NO) production, especially CUR and ISL. They were able to restore antioxidant enzyme activities both in vivo and in vitro. All treatments reduced inducible nitric oxide synthase (iNOS) expression compared to the untreated LPS control group. Behavioral assessments indicated that LPS impaired spatial and nonspatial memory, but treatments improved cognitive performance. Biomarker analyses revealed that ISL, HES, and CUR reduced the interleukin (IL)‐1β and nuclear factor erythroid 2‐related factor 2 (Nrf2) ratio in the hippocampus. Moreover, they decreased the level of caspase‐3 demonstrated by western blotting and tumor necrosis factor‐α (TNF‐α) level. Thereby they inhibited LPS elicited apoptosis. Likewise, their anti‐inflammatory effects were illustrated in the histopathological examination. Furthermore, they decreased the expression of amyloid‐β. The study reinforces the potential of these natural compounds as protective and therapeutic cost‐effective alternatives for managing neuroinflammation and neurodegeneration.

## 1. Introduction

The activation of glial cells and the production of proinflammatory mediators are features of neuroinflammation, a complicated immunological response that develops within the central nervous system (CNS). Aberrant or chronic neuroinflammation can cause serious neurological damage and is linked to a number of conditions, including neurodegenerative diseases and acute brain injuries, even while it protects against infections and accidents. Developing successful therapeutic strategies requires a comprehension of the causes and effects of neuroinflammation [1–3].

Furthermore, the gradual degeneration of neuronal cells is a hallmark of both acute and chronic neurodegenerative diseases. Huntington’s disease, Parkinson’s disease (PD), Alzheimer’s disease (AD), and amyotrophic lateral sclerosis (ALS) are a few examples of these diseases. In the course of many disorders, increased oxidative stress and neuroinflammation play an important part [4].

One protein whose impairment has been documented in many neurological disorders is the nuclear factor erythroid 2‐related factor 2 (Nrf2). Nrf2 is an important leucine‐zipper protein essential for the maintenance of redox homeostasis. According to reports, there is a decrease in Nrf2 levels in the hippocampi of AD patients, while its upregulation is a central part of neuroprotection in PD and ALS [5–7]. Upon exposure to oxidative stress, Nrf2 moves to the nucleus, where it binds to antioxidant response elements in the DNA, promoting the transcription of genes encoding antioxidant enzymes, including heme oxygenase 1, catalase, superoxide dismutase (SOD), and glutathione‐related enzymes, for example, glutathione‐S‐transferase (GST) and glutathione peroxidase (GPx). Nrf2 upregulation and nuclear translocation occur in response to inflammogens such as bacterial LPS and by several phytochemicals such as sulforaphane [8]. Additionally, nitric oxide (NO) is released in response to this inflammation. Overproduction of NO may aggravate neuroinflammation, leading to tissue damage and neuronal death [9].

Interestingly, the stimulation of brain microglial cells plays a central role in the pathogenesis of neuroinflammatory diseases. LPS activates toll‐like receptor 4 (TLR4), resulting in an acute inflammatory response, and thus, cultures of LPS‐challenged microglial cells serve as a good in vitro model of neuroinflammation [10, 11].

Through a variety of mechanisms, natural compounds have demonstrated potential in the treatment of neuroinflammation. They provide a multifaceted approach to treatment by focusing on important pathogenic aspects of neurological disorders, including oxidative stress, inflammation, and amyloid‐β accumulation. Flavonoids are important potential agents for neuroprotection through their antioxidant and anti‐inflammatory activities and their capability to act as signaling molecules [1, 12].

Among the natural compounds that might have this potential neuroprotective effect are hesperidin (HES), isoliquiritigenin (ISL), and curcumin (CUR) [13–20]. CUR is a polyphenolic molecule extracted from turmeric. It has attracted considerable interest due to its several medicinal benefits, including antioxidant, anti‐inflammatory, and anticancer activities [21–23].

HES is a notable flavonoid primarily located in citrus fruits, acknowledged for its diverse pharmacological attributes and possible positive health effects. This bioflavonoid demonstrates antioxidant, anti‐inflammatory, anticancer, and neuroprotective properties, rendering it significant for clinical and industrial uses. Its versatility transcends pharmaceuticals, since it is employed in food, cosmetics, and nutraceuticals [24, 25].

ISL is a bioactive chalcone molecule extracted from licorice, noted for its various pharmacological functions. Recent studies emphasized its potential therapeutic value in diverse diseases, including inflammation, cancer, and cardiovascular disorders [18, 26–29].

The current study aims to compare the in vitro and in vivo effects of CUR, HES, and ISL as protective agents and potential treatments against LPS‐induced neuroinflammation. It delves into the underlying mechanisms of these flavonoids, indicating their potential as cost‐effective natural adjuvant therapies for neuroinflammatory conditions. Notably, this research is unique, as it consolidates the effects and mechanisms of all three treatments within a single study, filling a gap in the existing literature that has not previously compiled these flavonoids together.

## 2. Materials and Methods

### 2.1. Chemicals

All chemicals were purchased from Sigma‐Aldrich (Germany). HES, ISL, and CUR were dissolved in DMSO (Roth, Germany) for cell culture experiments. Stock concentrations were as follows: ISL 1.35 M, HES 81.9 mM, and CUR 4.9 mM. Based on previous studies, the following dose ranges were selected: 6.25–50 μM ISL, 15–120 μM HES, 2.5–20 μM CUR, and 100 ng/mL LPS from *E. coli* (Sigma‐Aldrich, Germany) in PBS [30–32].

### 2.2. In Vitro Experiments

#### 2.2.1. Cell Viability Testing and NO Release

BV‐2 mouse microglial cells [RRID: CVCL_0182] were kindly provided by Dr. Anke Witting (Ulm University). They were cultivated in high‐glucose DMEM with L‐glutamine and 10% FBS (Serox, Germany) and kept in a humidified 5% CO_2_ atmosphere at 37°C. BV‐2 cells were cultured at a density of 2 × 10^5^ cells/mL in a 24‐well plate for the Griess assay and a 96‐well plate for the cytotoxicity assay. For the cytotoxicity assay, cells were seeded and incubated for 24 h. On the second day, cells were stimulated by adding the test compounds 1 h before adding LPS [33–36]. Cell viability was assessed by water‐soluble tetrazolium‐1 (WST‐1) assay using (ab155902WST‐1 Cell Proliferation agent; Abcam, Cambridge, UK) kit. The absorbance was measured at 450 nm using a Victor3 V plate reader (Perkin Elmer, Massachusetts, USA) [37]. For NO release, 24 h after exposure to the compounds, culture media were collected for Griess assay, in which nitrite concentration reflects the amount of NO produced. Under dark conditions, 500 μL of the culture supernatant was added to 250 μL of 1% sulfanilamide (Sigma‐Aldrich, Darmstadt, Germany) in 5% H_3_PO_4_ (Thermo Fisher Scientific, Inc., Waltham, MA, USA). After 5 min, 250 μL of 0.1% N‐(1‐Naphthyl) ethylenediamine dihydrochloride (Loba Chemie, Mumbai, India) was added to the reaction and incubated for 10 min at room temperature. Absorbance was measured at 540 nm, using a spectrophotometer (Jasco V‐630, Tokyo, Japan). Nitrite concentration was determined from the calibration curve constructed from NaNO_2_ standards (Riedel‐de Haën, Seelze, Germany). Relative NO production was calculated according to the following equation [38, 39]:

(1) relative NO production=nitrite concentration−mean nitrite from vehicle control cellsmean nitrite from LPS‐ treated cells−mean nitrite from vehicle control cells .

Data were fitted to the Hill equation, and dose–response curves were plotted to evaluate the effect of the treatments on nitrite production.

#### 2.2.2. Assessment of Antioxidant Enzyme Activities and Nrf2 Levels

BV‐2 cells (4 × 10^5^ cells/mL) were cultured in 6‐well plates, pretreated with the test compounds at the selected concentrations, and LPS was added after 1 hour. Twenty‐one hours later, PBS was used to wash the cells twice, and then they were stored on ice shortly before harvesting them. Sucrose mannitol buffer (210 mM mannitol and 70 mM sucrose) was used for cell lysis. Next, nuclear and cytoplasmic fractions were separated by centrifugation at 1000 × *g* for 10 min at 4°C and then at 10,000 × g for 10 min at 4°C, and the protein content of each fraction was assessed using the Bradford assay [40]. The nuclear fraction was used to measure nuclear Nrf2 levels, while the cytoplasmic fraction was used to assess enzymatic activities and the cytosolic Nrf2 levels.

Total SOD activity was assessed colorimetrically by the total superoxide dismutase activity assay kit (Elabscience, Houston, Texas, USA) that utilizes xanthine oxidase and WST‐1 to assess SOD activity [41]. GPx activity was assessed using a glutathione peroxidase activity colorimetric assay kit (Biovision, Milpitas, CA, USA). GPx activity was assessed from the rate of oxidation of reduced glutathione (GSH) to the oxidized form of glutathione (GSSG) and the reduction of cumene hydroperoxide. GSSG reduction to GSH requires NADPH as an electron source. The reduction in NADPH concentration, measured at 340 nm, is proportional to GPx activity [42]. GST activity was measured using the ab65326 GST Activity assay kit (Abcam, Cambridge, UK), which measures the rate of formation of the reaction product of GSH with GST substrate, 1‐chloro‐2,4‐dinitrobenzene, at 340 nm [43]. The nuclear and cytoplasmic concentrations of Nrf2 protein were measured in BV‐2 cell lysates using a mouse Nrf2 ELISA kit (Cusabio, Houston, Texas, USA). The manufacturer’s guidelines were followed in all assays.

### 2.3. In Vivo Experiments

#### 2.3.1. Animals

Adult male Swiss albino mice (25–30 g) were purchased from the Modern Veterinary (Giza, Egypt). They were allowed to acclimatize in the animal facility at October University for Modern Sciences and Arts (MSA University) for one week, where they were exposed to controlled environmental conditions (23°C ± 2°C and a 12 h/12 h dark/light cycle). They had free access to standard chow pellets and water. Guidelines from MSA University′s Ethics Committee (PH1/EC1/2023PD) and the National Institutes of Health’s Guide for Care and Use of Laboratory Animals (Publication No. 8523, amended 1985) were followed when treating the animals. All animal experiments have been conducted in accordance with ARRIVE criteria.

#### 2.3.2. Experimental Design

**FIGURE 1:**
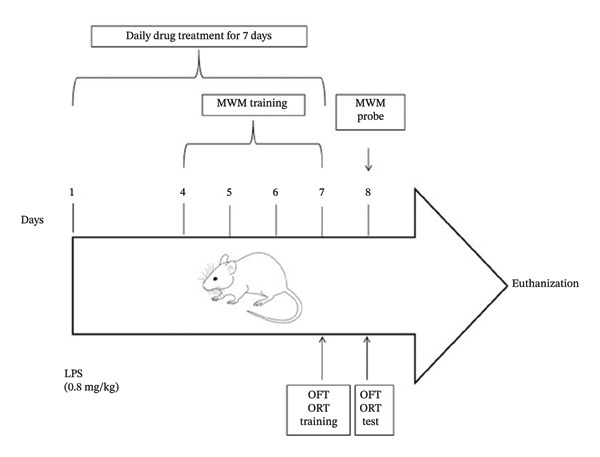
Schematic diagram of the experimental design. A single dose of 0.8 mg/kg LPS was injected ip on day one in all groups except the control group. The test compound was injected ip daily on Days 1–7 in all treatment groups. MWM: Morris water maze, OFT: open field test, ORT: object recognition test.

The mice were randomly divided into five groups (*n* = 21/group): control, LPS, CUR, HES, and ISL. On the first day, the control group received a single intraperitoneal (ip) injection of PBS, followed by daily ip injections of 0.2 mL of 50% DMSO in PBS for six consecutive days. The LPS group received an ip injection of 0.8 mg/kg LPS on Day 1, with subsequent daily ip injections of 0.2 mL of 50% DMSO in PBS for the next 6 days. In the treatment groups, mice received an initial ip injection of 0.8 mg/kg LPS on Day 1, followed by daily ip injections of the test compounds: 50 mg/kg CUR, 100 mg/kg HES, and 25 mg/kg ISL [44–48] as shown in Figure 1. The injection plan of DMSO and compounds started 3 h after the initial PBS or LPS dose. Mice were sacrificed after the completion of behavioral testing. After excision of their brain cortexes, hippocampi were dissected for the subsequent biochemical investigations. The hippocampi and brain cortexes of every 3 animals were pooled together and considered as one sample.

#### 2.3.3. Immunohistochemistry and Determination of iNOS

Brain tissue sections were sliced onto adhesive slides, then deparaffinized and rehydrated in distilled water. Next, a heat‐induced epitope retrieval step was performed. The tissue sections were incubated with primary anti‐iNOS antibody (dilution 1:100, Santa Cruz, Inc., USA) for 2 h at room temperature. After washing, an HRP‐labeled detection kit (BioSB, USA) was used according to the manufacturer’s instructions to develop the color. Mayer’s hematoxylin served as a counterstain. Negative control slides were prepared by omitting the incubation with primary antibody. Positive expression was quantified as the percentage of area representing each group. The (iNOS) protein was determined using NOS2/iNOS antibody (C‐11): sc‐7271 [49].

#### 2.3.4. Behavioral Experiments

Training on the Morris water maze (MWM) took place on Days 4–7. On the 7^th^ day, mice were trained on the open field test (OFT) and the object recognition test (ORT). On Day 8, the probe test, the OFT, and the ORT were performed. The mice behaviors were video recorded for subsequent analysis by a blinded investigator.

The OFT was conducted to exclude any effects of LPS on the motor activity of mice. The mean ambulation, rearing, and grooming frequencies were recorded by a blinded investigator using hand‐operated counters within 3 minutes [50]. The ORT was used to evaluate nonspatial memory in mice. Following the OFT, the mice were trained on the ORT. During the training phase, the mice were permitted to examine two items that are identical and placed at opposite ends of the open‐field arena. After 24 h, during the choice trial, one of the acquainted items (*F*) was substituted with a novel, distinct item (*N*). Item identification was measured by counting the total amount of cases the animals sniffed *F* or *N*, and the preference index (PI) was calculated using the following equation [51]:

(2) PI=NN+F×100.

The MWM task was conducted to evaluate the spatial memory of the animals. The mean escape latency (MEL) was measured over the four training days during the acquisition phase. In the probe test, the percentage of time the mice stayed in the target quadrant compared to the other quadrants was calculated and reported as percent quadrant time (*Q*) [52].

#### 2.3.5. Quantification of Hippocampal Nrf2, Interleukin (IL)‐1β, and Caspase‐3

Nuclear and cytoplasmic proteins were extracted using the NE‐PER nuclear and cytoplasmic protein extraction kit (Thermo Scientific, Waltham, MA, USA). Hippocampal Nrf2 was assayed using a mouse Nrf2 ELISA kit (Aviva Systems Biology, San Diego, CA, USA); hippocampal IL‐1β content using a mouse IL‐1β ELISA kit (Cohesion Biosciences, London, UK); and caspase‐3 content using a mouse caspase‐3 ELISA kit (Cusabio Biotech, Wuhan, China). All procedures were done in accordance with the instructions of the manufacturers. Protein concentrations were assessed using the Bradford assay to estimate the protein concentration per total gram of protein [40].

#### 2.3.6. Determination of the Expression of Procaspase and Caspase‐3

Procaspase3 and caspase‐3 expression in the brain cortexes of all groups were determined using western blotting using the Bio‐Rad gel electrophoresis system, Mini‐PROTEAN Tetra Cell by Mini Trans‐Blot, and Bio‐Rad ChemiDoc gel documentation imaging system according to literature [53]. Caspase‐3 and GAPDH antibodies were obtained from Santa Cruz (USA), and rabbit anti‐mouse IgG (H + L) secondary antibody, HRP‐conjugated secondary antibody, (Invitrogen, Thermo Scientific, USA). Results were normalized to GAPDH, then to control. The percentage of decrease in the caspase/procaspase ratio was calculated relative to the LPS ratio group.

#### 2.3.7. Determination of Amyloid‐β and Tumor Necrosis Factor‐α (TNF‐α)

Brain tissues from different groups were used to assess the amyloid‐β accumulation and TNF‐α using the mouse Ab1‐42 (amyloid‐β peptide 1‐42) ELISA kit (ELK10615‐ELK Biotechnology, USA) and the mouse TNF‐α ELISA kit (ELK1387‐ELK Biotechnology, USA). All procedures were done in accordance with the manufacturer’s guidelines.

#### 2.3.8. Histopathological Examination

The brain specimens from each group were collected and fixed in 10% neutral buffered formalin. They were then embedded in paraffin and sectioned, with a 3–5 μm thickness, after being processed through grades of ethanol and xylene. The sections were stained with hematoxylin and eosin (H&E) and examined using a light microscope (DM4 B, Leica, Germany) [54].

#### 2.3.9. Assessment of Hippocampal Malondialdehyde (MDA) Content, SOD, GST, and GPx Activities

Hippocampal MDA content and SOD activity were assessed colorimetrically using lipid peroxide (malondialdehyde) and superoxide dismutase colorimetric assay kits (Biodiagnostic, Giza, Egypt). GST and GPx activities were determined using commercial kits (Biodiagnostic, Giza, Egypt). All procedures were done in accordance with the manufacturer’s guidelines. Protein concentrations were measured using the Bradford assay [40] and are used to estimate the MDA content and enzyme activities per total gram of protein.

### 2.4. Statistical Analysis

The analysis of data was performed using GraphPad Prism software 6 (GraphPad Prism Software Inc., USA). Differences between groups were assessed via one‐way ANOVA, followed by Tukey’s post hoc test. A *p* value of < 0.05 was considered statistically significant.

## 3. Results

### 3.1. In Vitro Assays

#### 3.1.1. Cell Viability Testing and NO Release

**FIGURE 2:**
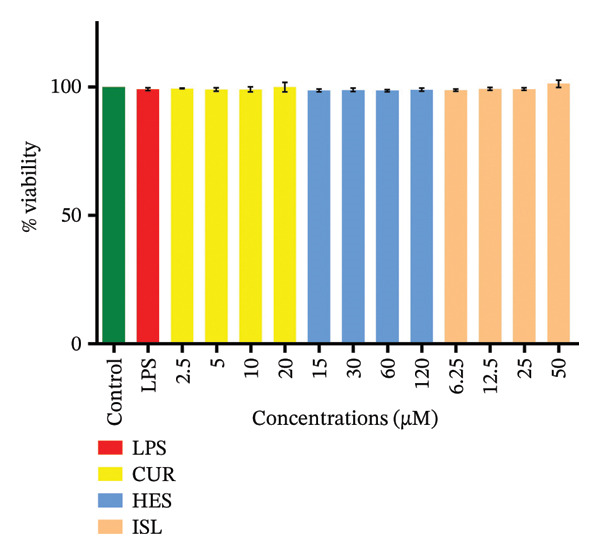
Viability of BV‐2 cells following treatment with LPS and various doses of the tested compounds in comparison to untreated cells.

**FIGURE 3:**
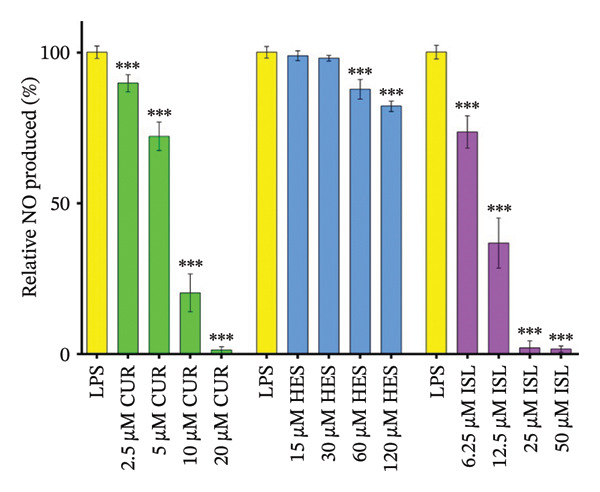
Impact of treatments on relative NO production in LPS‐treated BV‐2 cells. Except for HES, all treatments caused a dose‐dependent reduction of LPS‐elicited NO production. For dose–response curves, data were fitted to the Hill equation. IC_50_ values were 6.1 ± 0.8 and 10.8 ± 1.5 μM for CUR and ISL, respectively. Results are displayed as means ± SDs. ^∗∗∗^ *p* < 0.001 versus the LPS‐treated cells using one‐way ANOVA.

Before assaying the effect on NO, a viability assay was performed using LPS and different concentrations of the tested compounds. Both the LPS and the treatments did not imapct BV‐2 cell viability, as shown in Figure 2. LPS treatment caused a 24.7‐fold increase in nitrite production. ISL and CUR caused a dose‐dependent reduction of LPS‐induced NO production with IC_50_ values of 10.8 ± 1.5 μM and 6.1 ± 0.8 μM, respectively, as shown in Figure 3.

#### 3.1.2. Assessment of Antioxidant Enzyme Activities and Nuclear/Cytoplasmic Nrf2 Ratio

In the current study, LPS treatment of BV‐2 cells significantly decreased GPx, GST, and SOD activities compared to the vehicle‐treated control (*p* < 0.001). While CUR and ISL alleviated the decrease in GPx activities after LPS treatment, HES was capable of enhancing GPx activity significantly (*p* < 0.05) compared to the LPS group. Moreover, HES and CUR significantly increased activity of GST compared to LPS‐treated cells at *p* < 0.001. On the other hand, ISL and CUR enhanced SOD activity compared to the untreated LPS group at (*p* < 0.001). LPS caused a significant decrease in the nuclear/cytoplasmic Nrf2 ratio compared to vehicle‐treated controls at *p* < 0.001. On the other hand, all the tested compounds attenuated the LPS‐mediated effect significantly compared to the LPS group (*p* < 0.001 and *p* < 0.01) as shown in Table 1.

**TABLE 1:** Effect of CUR, HES, and ISL on activities of GPX, GST, SOD, and the ratio of nuclear/cytoplasmic Nrf2 levels in LPS‐treated BV‐2 cells.

	Control	LPS	CUR	HES	ISL
GPx (% of control)	100.0 ± 1.20	88.3 ± 1.82^###^	91.9 ± 1.37	95.8 ± 2.78^∗^	93.2 ± 2.11
GST (% of control)	100.01 ± 0.27	93.57 ± 0.24^###^	97.17 ± 0.17^∗∗∗^	98.09 ± 0.14^∗∗∗^	94.29 ± 0.29
SOD (% of control)	100.00 ± 0.05	96.75 ± 0.07^###^	98.33 ± 0.07^∗∗∗^	96.67 ± 0.07	99.65 ± 0.07^∗∗∗^
Nuclear/cytoplasmic NRF2	100 ± 3.63	60 ± 1.92^###^	91 ± 3.55^∗∗∗^	87 ± 4.69^∗∗∗^	74 ± 3.64^∗∗^

*Note:* All values are presented as means ± SEMs. 20 µM CUR, 120 µM HES, and 25 µM ISL were used. Duplicates from 3 independent experiments were carried out.

^###^Significance versus the control group *p* < 0.001.

^∗^Significance *p* < 0.05.

^∗∗^Significance *p* < 0.01.

^∗∗∗^Significance *p* < 0.001 versus the LPS‐treated BV‐2 cells.

### 3.2. In Vivo Experiments

#### 3.2.1. Immunohistochemistry and Determination of iNOS

The expression of iNOS was significantly higher in the LPS group than in the control group. The CUR group had the lowest iNOS expression, whereas the ISL and HES groups also had significant declines in iNOS expression with no discernible differences between them compared to LPS group, as shown in Figures 4(a) and 4(b).

FIGURE 4(a) Immunohistochemistry of brain cortex to determine iNOS expression. (A) Photomicrograph of cerebral cortex, control group showing negative iNOS expression. (B) Photomicrograph of cerebral cortex, LPS group showing intense iNOS expression. (C) Photomicrograph of cerebral cortex, CUR‐treated group showing limited iNOS expression. (D) Photomicrograph of cerebral cortex, ISL‐treated group showing moderate iNOS expression. (E) Photomicrograph of cerebral cortex, HES‐treated group showing moderate iNOS expression. All Images were obtained in duplicates (b) Quantification of iNOS as an area percentage. Data are presented as mean ± SEM. Significant difference is considered at ^∗^ *p* < 0.05, ^∗∗^ *p* < 0.01, ^∗∗∗^ *p* < 0.001, ^∗∗∗∗^ *p* < 0.0001.

**Figure:**
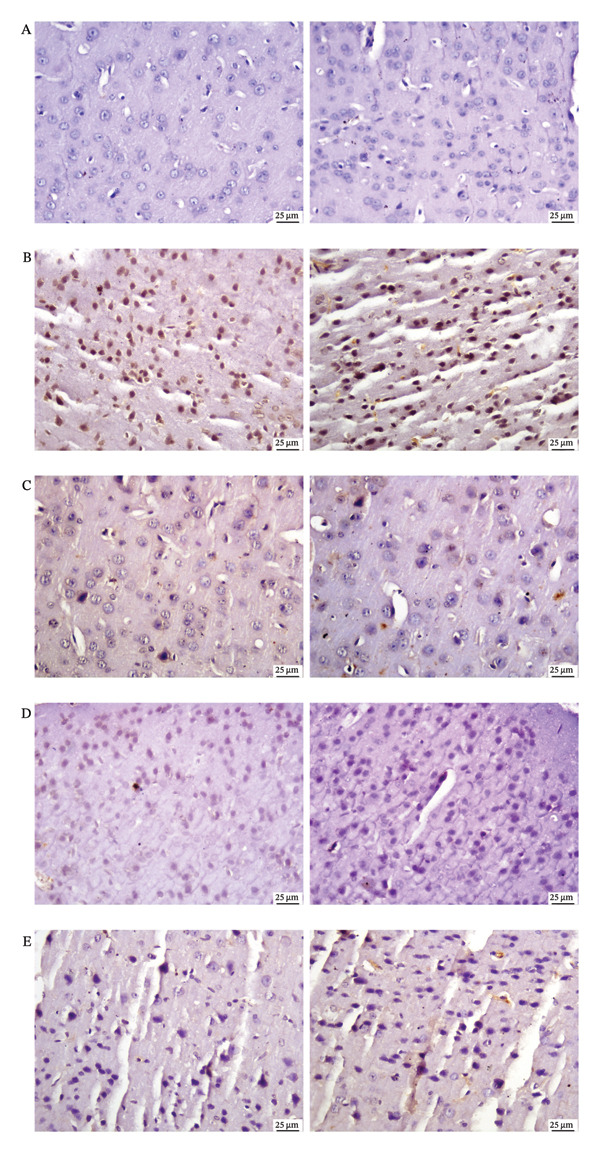
(a)

**Figure:**
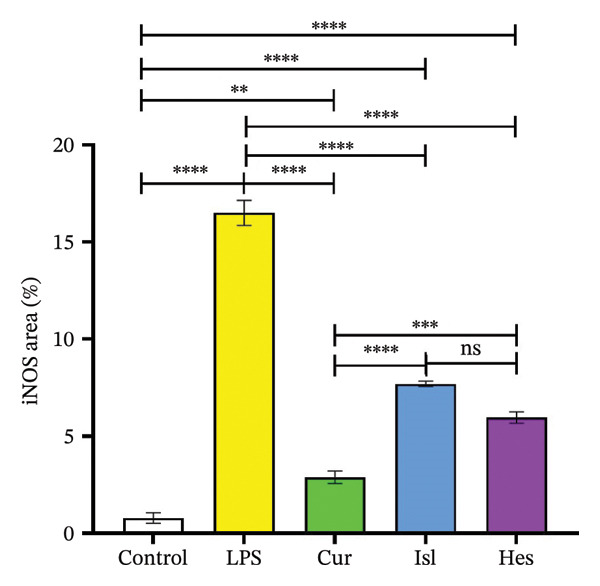
(b)

#### 3.2.2. Behavioral Experiments

HES, ISL, and CUR improved hippocampal‐dependent memory tasks. The OFT confirmed that the LPS treatments had no effect on the motor system of the mice. The LPS group showed impaired spatial memory as evidenced by the MWM task. LPS significantly increased the MEL time on Day 7 to 148 ± 2.6% and decreased percent quadrant time (Q) significantly to 55.4% ± 4.1% of the control group. Treatments reversed the LPS effect in the MWM. CUR, HES, and ISL treatments significantly reduced MEL time on day seven by 33%, 31%, and 28%, respectively, and prolonged Q by 69%, 87%, and 83% relative to the LPS group, as shown in Figures 5(a) and 5(b). In the ORT, LPS significantly reduced PI to 50% ± 3% in contrast with the control group. Treatment using CUR, ISL, and HES significantly ameliorated the nonspatial memory and resulted in a significant elevation of PI% by 46%, 46%, and 53% compared to the LPS group, as shown in Figure 5(c).

FIGURE 5Behavioral assessments. Impact of the tested compounds on memory in LPS‐treated mice. (a) MEL evaluated spatial memory in the MWM during training days. (b) The Probe test conducted on the final day of the experiment. (c) The discrimination index assessing non‐spatial memory in the object recognition test. All treatments alleviated the effects of LPS on the memory of mice. ^#^ *p* < 0.01 in contrast with the control group; ^∗^ *p* < 0.05, ^∗∗^ *p* < 0.01 in contrast with the LPS group.

**Figure:**
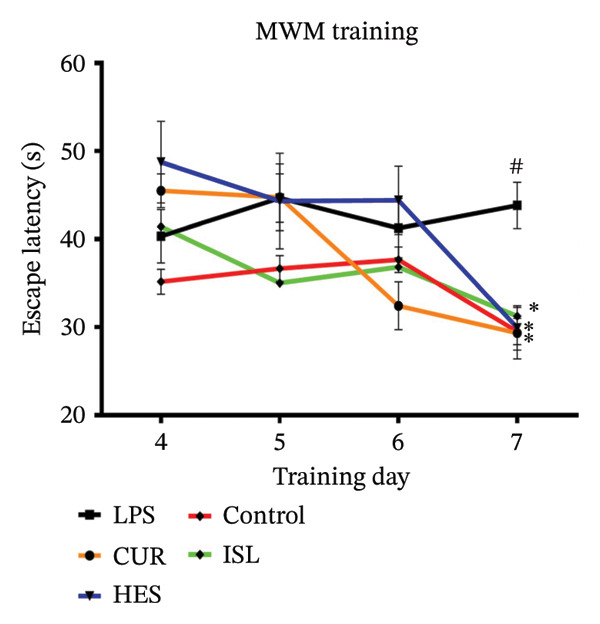
(a)

**Figure:**
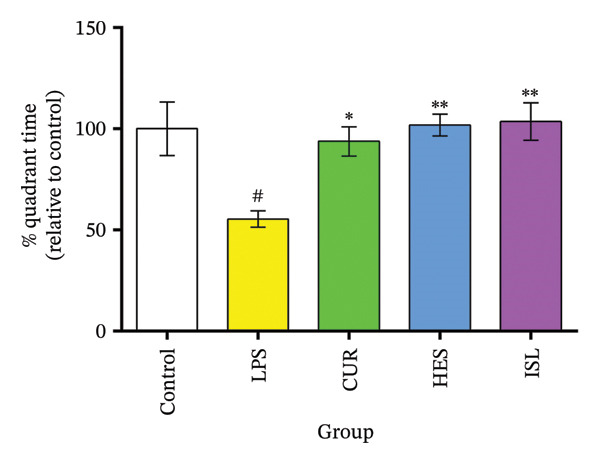
(b)

**Figure:**
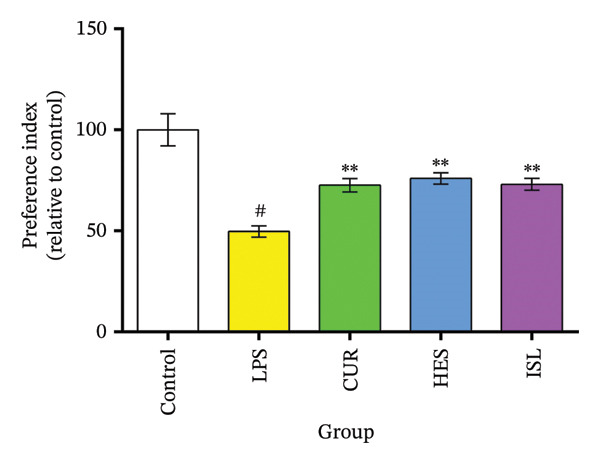
(c)

#### 3.2.3. Quantification of Hippocampal MDA Content, SOD, GST, and GPx Activities, Nrf2ratio, IL‐1β, and Caspase‐3 Levels

In this study, LPS treatment increased oxidative stress in mice, as shown by the significant rise in hippocampal MDA in the LPS group to 144.6% ± 10.3% of the control mice. Furthermore, mice treated with LPS exhibited a significant decrease in hippocampal GST, SOD, and GPx activities to 38.3% ± 4.1%, 73.0% ± 4.5%, and 59.8% ± 3.6% of the control, respectively. HES and ISL treatments reduced MDA content by 29.5% and 23.9%, respectively, relative to LPS‐treated mice and enhanced hippocampal GST activity and normalized it to control level. HES and ISL capacities to enhance GST activity were higher than CUR, elevating GST activity by 150% for both HES and ISL, compared to 88.5% by CUR, compared to the LPS‐treated group. All treatments reversed the effect of LPS on hippocampal SOD, increasing SOD activity by 37.7%, 24%, and 30.4% for HES, ISL, and CUR, respectively. In contrast, only HES produced influenced GPx activity, increasing it by 32%, as shown in Figure 6(a). While LPS elevated hippocampal IL‐1β and caspase‐3 content to 211% ± 18% and 186% ± 9%, respectively, treatment by HES and ISL normalized IL‐1β and caspase‐3 contents, reducing IL‐1β by 53.2% and 46.7% compared to 60.0% by CUR, and caspase‐3 by 37.6% and 42.8% compared to 28.8% by CUR relative to the LPS group, as shown in Figure 6(b).

FIGURE 6Biochemical impacts of treatments on the hippocampi of mice. (a) Impact of the tested compounds on oxidative stress markers in the hippocampus. (b) Impact of the tested compounds on hippocampal IL‐1β and caspase‐3 levels. (c) Nuclear/cytoplasmic Nrf2 ratios. The results are presented as means ± SEM. ^#^ *p* < 0.001 compared to the control group; ^∗^ *p* < 0.05, ^∗∗^ *p* < 0.01, ^∗∗∗^ *p* < 0.001 compared to the LPS group. Investigation was performed via one‐way ANOVA.

**Figure:**
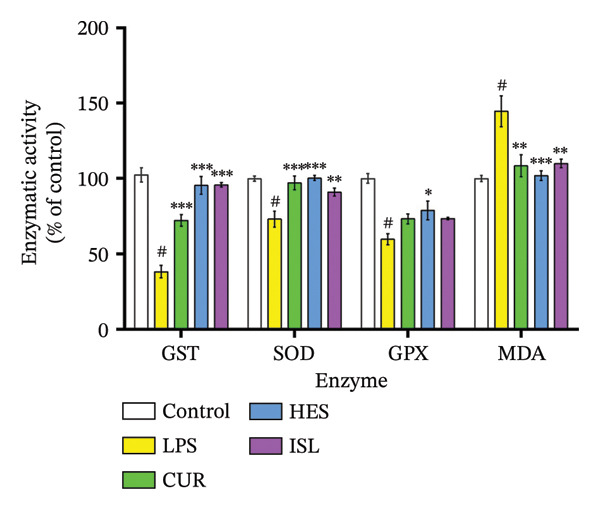
(a)

**Figure:**
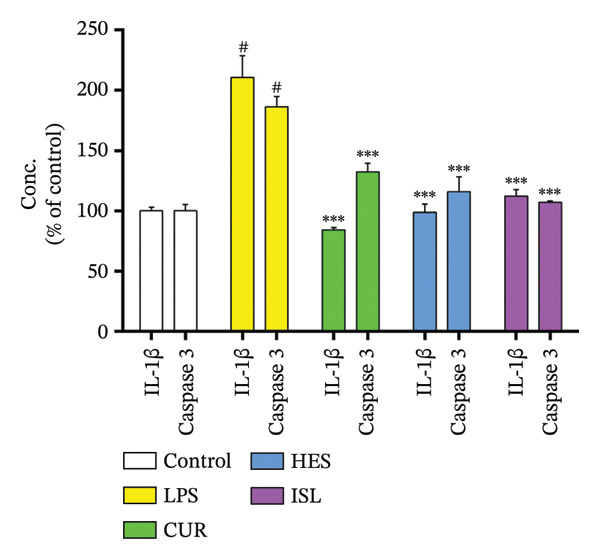
(b)

**Figure:**
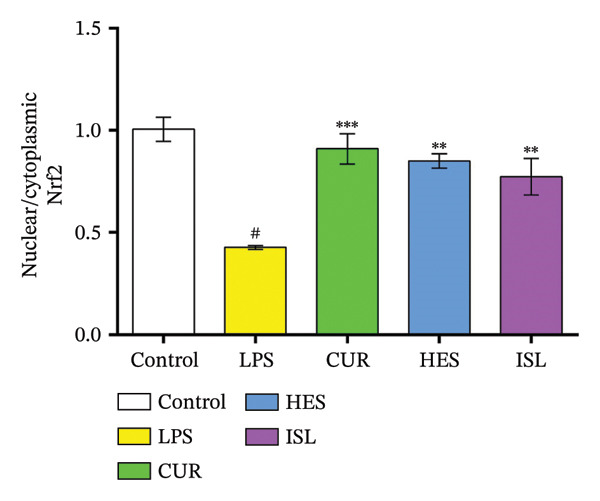
(c)

LPS significantly lowered the Nrf2 nuclear/cytoplasmic ratio in contrast with the control group. All treatments elevated the nuclear/cytoplasmic Nrf2 ratio back to the control level, as shown in Figure 6(c).

#### 3.2.4. Determination of the Expression of Procaspase‐3 and Caspase‐3 Through Western Blotting

The effects of LPS and different treatments on procaspase, caspase‐3, and GAPDH expression are shown in Figure 7 and Table 2. The CUR group exhibited a 65.4% decrease in the level of caspase‐3 compared to the LPS. The HES group exhibited a 69.9% decrease in the level of caspase‐3 compared to LPS. The ISL group exhibited a 97.3% decrease in the level of caspase‐3 compared to the LPS.

FIGURE 7(a) Western blotting results of procaspase‐3 in control, LPS, and different treatment groups. (b) Western blotting results of caspase‐3 in control, LPS, and different treatment groups. (c) Western blotting results of GAPDH in control, LPS, and different treatment groups.

**Figure:**
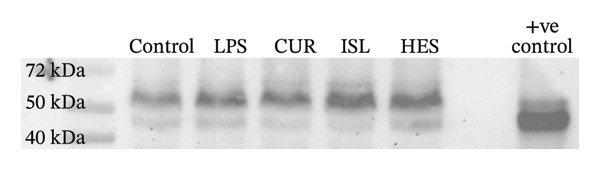
(a)

**Figure:**
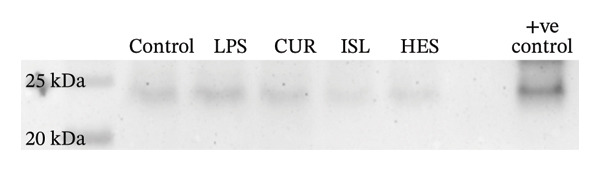
(b)

**Figure:**
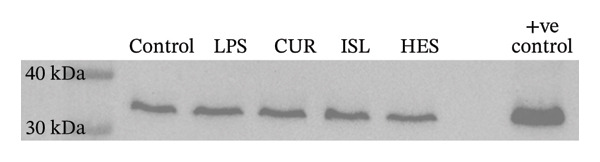
(c)

**TABLE 2:** Effect of CUR, HES, and ISL on the caspase and procaspase ratio in LPS‐treated brain cortex.

	Control	LPS	CUR	HES	ISL
Caspase/Procaspase ratio	1	2.4	0.85	0.74	0.066

#### 3.2.5. Determination of Amyloid‐β and TNF‐α

The expression of amyloid‐β was significantly different in the control versus the LPS group. All treatments displayed a significant decrease in amyloid‐β compared to LPS groups, as shown in Table 3. There was a significant difference in the levels of TNF‐α between the control and LPS groups. All treatment groups expressed a significant decrease compared to the LPS group, as shown in Table 3.

**TABLE 3:** Effect of CUR, HES, and ISL on expression of amyloid‐β and the TNF‐α levels in LPS‐treated brain cortexes.

	Control	LPS	CUR	HES	ISL
Amyloid‐β pg/mg tissue	34.2 ± 5.8^∗∗^	74.4 ± 8.4	39.9 ± 5.4^∗∗^	35.3 ± 4.5^∗∗^	41.1 ± 5.7^∗∗^
TNF‐α pg/mg tissue	85.2 ± 9.3^∗∗∗^	353.1 ± 15.6	21.9 ± 4.1^∗∗∗^	66.13 ± 8.6^∗∗∗^	132.0 ± 11.2^∗∗∗^

*Note:* All values are presented as means ± SD. Duplicates from 3 independent experiments were carried out.

^∗^Significance at *p* < 0.05.

^∗∗^Significance at *p* < 0.01.

^∗∗∗^Significance at *p* < 0.001 versus the LPS‐treated animals.

#### 3.2.6. Histopathological Examination

Microscopic images of brain tissue showed normal histological structure of the cerebral cortex in the control group, as shown in Figures 8(a) and 8(b). Mice in the CUR group revealed apparently normal histological structure with mild neuronal degeneration and vascular congestion, as shown in Figures 8(c) and 8(d). On the other hand, the group that received LPS showed marked pathological alterations. Pyramidal cells of the brain cortex exhibited severe neuronal degeneration and neurophagia associated with astroglial and microglial reactions. The degenerated neurons appeared with pyknotic nuclei and eosinophilic cytoplasm, as shown in Figures 8(e) and 8(f). Minute foci of hemorrhage were observed in the deep cortex in some examined sections. As depicted in Figures 8(g) and 8(h), mice in the ISL group had an ameliorative effect; the brain cortex exhibited mild to moderate degeneration of neurons with neurophagia. Poor improvement was observed in the HES group with moderate to severe neuronal degeneration associated with glial reactions and vascular congestion, as shown in Figures 8(i) and 8(j).

FIGURE 8(a) Photomicrograph of brain, cerebral cortex, control group showed normal histological structure (H&E), 200x. (b) Photomicrograph of brain, cerebral cortex, control group, higher magnification showed normal histological structure (H&E), 400x. (c) Photomicrograph of brain, cerebral cortex, CUR group showed cerebral vascular congestion (arrow) (H&E), 200x. (d) Photomicrograph of brain, cerebral cortex, CUR group, higher magnification, showed mild cerebral vascular congestion (H&E), 400x. (e) Photomicrograph of brain, cerebral cortex, Induction LPS group showed numerous dark degenerating neurons and neuronophagia (arrow) (H&E), 200x. (f) Photomicrograph of brain, cerebral cortex, induction group (LPS); higher magnification showed numerous dark degenerating neurons and neuronophagia (arrow) (H&E), 400x. (g) Photomicrograph of brain, cerebral cortex, ISL group showed moderate neuronal degeneration (arrow) (H&E), 200x (h) Photomicrograph of brain, cerebral cortex, ISL, higher magnification showed moderate neuronal degeneration (H&E), 400x. (i) Photomicrograph of brain, cerebral cortex, HES group, showed numerous dark degenerating neurons and neuronophagia (arrow) (H&E), 200x. (j) Photomicrograph of brain, cerebral cortex, Hes group, higher magnification showed dark degenerating neurons and neuronophagia (arrow) (H&E), 400x.

**Figure:**
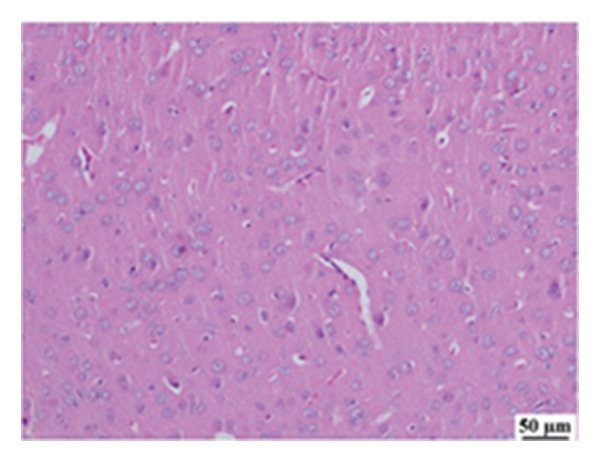
(a)

**Figure:**
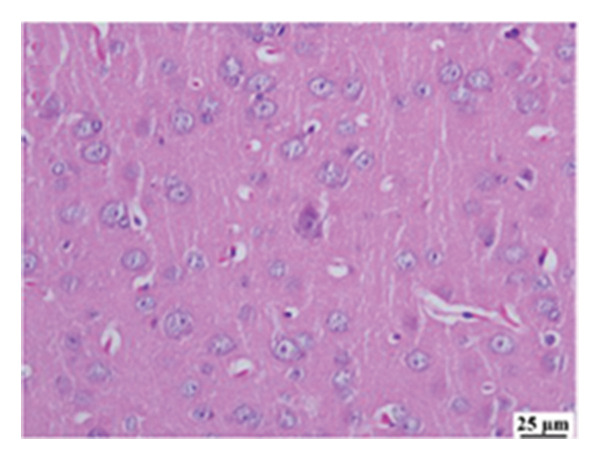
(b)

**Figure:**
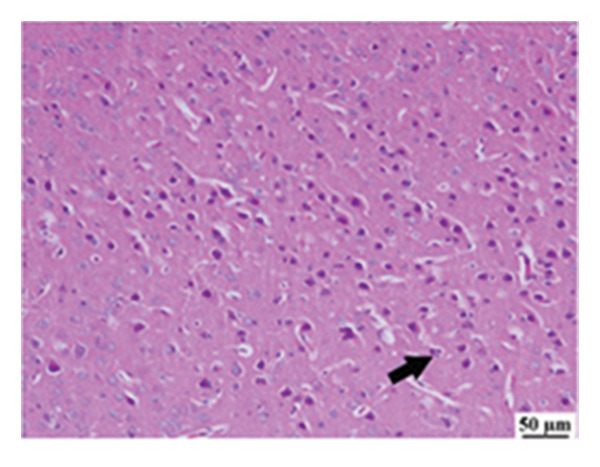
(c)

**Figure:**
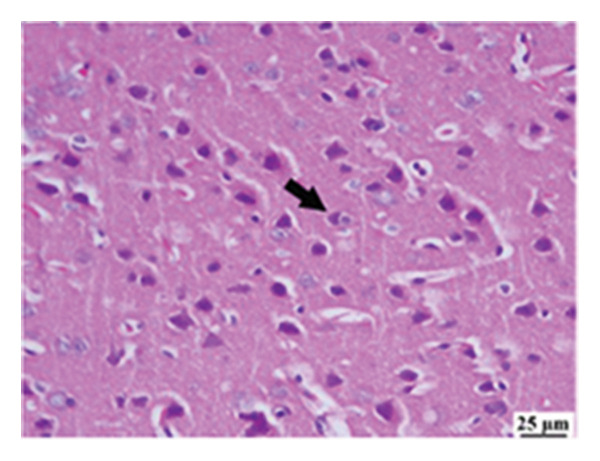
(d)

**Figure:**
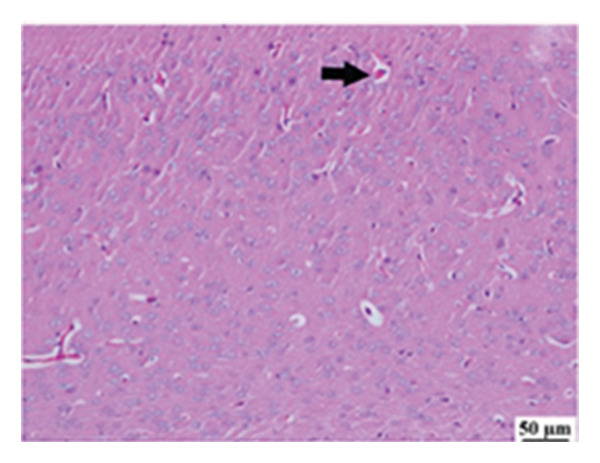
(e)

**Figure:**
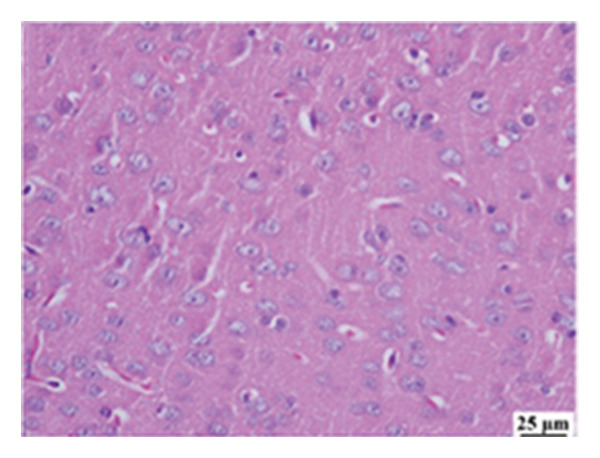
(f)

**Figure:**
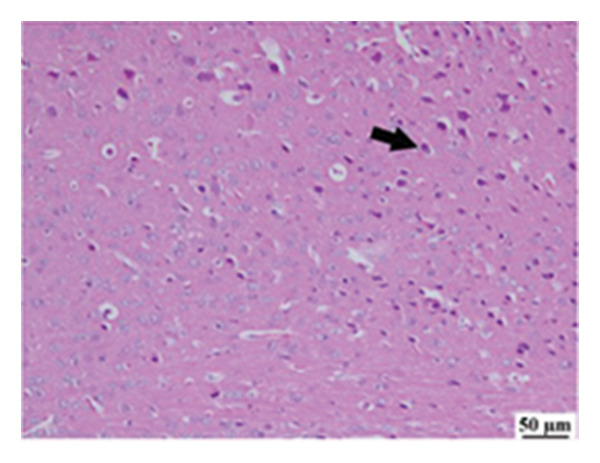
(g)

**Figure:**
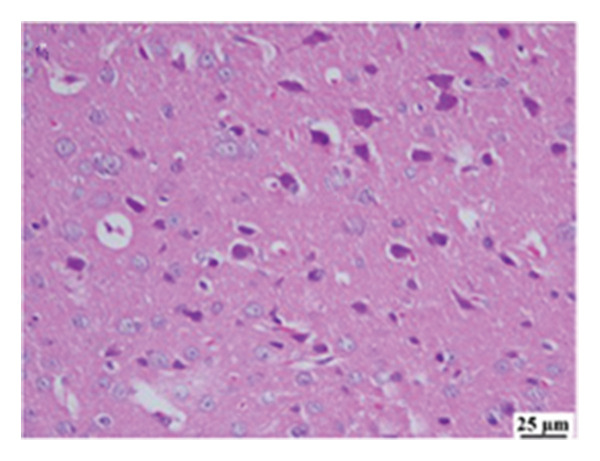
(h)

**Figure:**
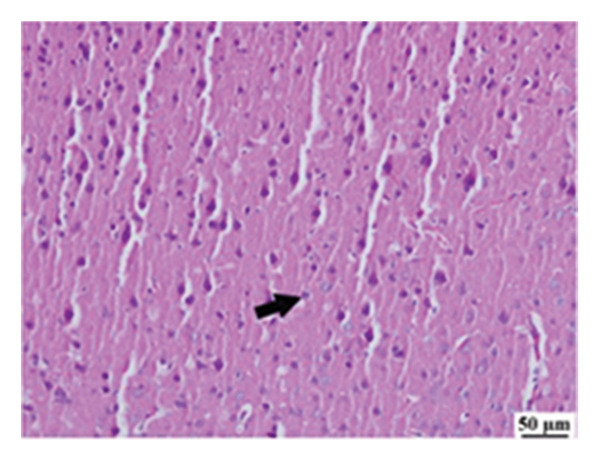
(i)

**Figure:**
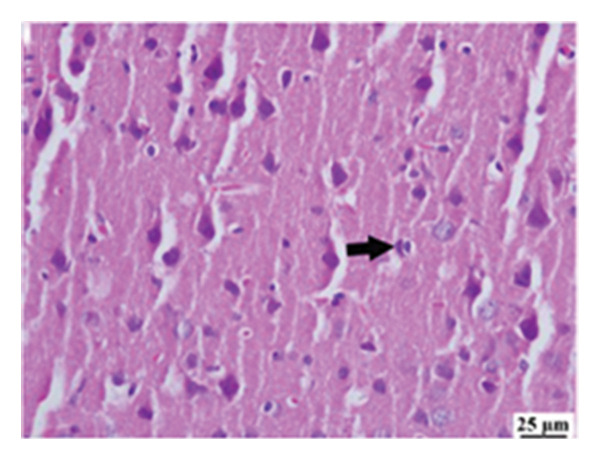
(j)

## 4. Discussion

Neuroinflammation is a hallmark of several neurodegenerative diseases, and its early alleviation is the most promising treatment approach. One possible route is the early use of natural compounds with neuroprotective effects. These would slow down both the oxidative and inflammatory processes, which start early before the clinical symptoms are manifested. In the current investigation, we demonstrate that ISL, HES, and CUR could attenuate LPS‐stimulated neuroinflammation and oxidative stress in vitro as well as in vivo.

The LPS as well as the different doses of the treatments used were first tested for their effect on the cell viability, and all treatments proved safe. Therapy of the cells with LPS alleviated the NO production. In vitro, pretreatment of BV‐2 cells with ISL and CUR inhibited NO production in a dose‐dependent manner through the inhibition of the NF‐κB pathway, therefore reducing the transcription of iNOS [30, 55, 56]. Furthermore, CUR was shown to inhibit NO production through its inhibitory effect on c‐Jun N‐terminal kinase (JNK) and p38 along with NF‐κB [32]. Although HES has been reported to attenuate LPS‐induced iNOS expression and subsequent NO production in the mouse macrophages RAW 264.7 cells by obstructing the NF‐κB pathway [57], our findings in the BV‐2 cells were not the same. However, the effect induced by HES treatment was further illustrated when determining iNOS expression in mouse brain. Thereby, all treatments were successful in decreasing expression of iNOS compared to the LPS group [58–60].

In the animal model, different doses of the drugs were used. The aim was to mimic the normal dose an individual would take per serving. HES was reported to be consumed at a dose of 43.7 mg/100 mL orange juice [61], CUR was consumed in some countries daily at an average of 2.7–14 mg [62], and ISL at a safe dose of around 20–30 mg [63, 64].

The variation between in vitro and in vivo NO results after HES treatment can further be attributed to the fact that BV‐2 cells were exposed to LPS for a shorter interval than mice. Correa et al. reported a time‐dependent effect of LPS on neonatal mice brains, where the brain cells were able to counteract LPS detrimental effects in the first 24 h, but not in an interval of 72 h [65]. Furthermore, Lund et al. found that the gene expression profile after LPS’s stimulation was the same in BV‐2 cells compared to primary cultured microglial cells, with the effect evoked by LPS application being weaker in BV‐2 cells [66]. HES, ISL, and CUR show different antioxidant properties in vivo compared to in vitro due to the complex biological interactions and metabolic processes present in living organisms. Moreover, in vitro investigations, such as those employing BV2 microglial cells, provide regulated settings for measuring direct cellular reactions but do not account for more extensive systemic interactions [67, 68].

To exclude any possible deficits in neuromotor functions that might affect the performance of mice during the behavioral tasks, the OFT was conducted, and no significant variations were observed between the groups across any of the parameters. Spatial and nonspatial memories of mice deteriorated after LPS treatment, thus validating the model. Treatment of the mice with the tested compounds improved both the spatial and nonspatial memories, indicating the capability of these compounds to cross the blood–brain barrier and affect the brain, as reported by others [28, 67, 69].

To explain this decline in memory and improvement after treatment, several biomarkers were measured. IL‐1β was evaluated alongside TNF‐α, amyloid‐β, and caspase‐3. While LPS increased IL‐1β and caspase‐3, TNF‐α and amyloid‐β, treatments reduced these biomarkers. It has been demonstrated that cytokines like IL‐1β and TNF‐α exhibit dual roles in brain physiology, serving as both enhancers of cognitive function and agents of cognitive decline. This duality is especially apparent in diseases such as inflammation and AD, where these cytokines may intensify neurodegenerative processes [70–73]. Amyloid‐β isoforms, as memory blood‐based biomarkers, are essential for the diagnosis and monitoring of AD. These biomarkers indicate amyloid processing, neuroinflammation, and synaptic dysfunction, which are pivotal to AD pathophysiology [74–76].

In the present study, ISL, HES, and CUR suppressed neuroinflammation in vivo, reducing levels of LPS‐induced IL‐1β in mice hippocampi to pretreatment levels. This could be attributed to HES’s and CUR’s positive effect on the anti‐inflammatory miRNA‐132 [77, 78]. The miRNA‐132 inhibits IL‐1 receptor‐associated kinase 4 (IRAK4), an effector protein of TLR‐2/4 and a regulator of NF‐κB, leading to a decrease in IL‐1β and IL‐6 levels [79, 80]. Downregulation of miRNA‐132 has been postulated to cause increased tau phosphorylation in AD [81]. The suppression of neuroinflammation was further demonstrated by the decrease in iNOS expression in all treatment groups compared to LPS.

Interestingly, in the study of neurodegenerative diseases such as AD, the interaction between TNF‐α and amyloid‐β in LPS‐induced neuroinflammation is essential. While amyloid‐β is associated with the pathophysiology of the disease, TNF‐α is a proinflammatory cytokine important in neuroinflammation [71, 82]. These inflammatory processes may be reduced by natural substances with anti‐inflammatory and neuroprotective qualities, such as HES, CUR, and ISL. By altering the TLR4/NF‐κB pathway, HES lowers TNF‐α and IL‐1β levels and enhances cognitive performance [71, 82]. Interestingly, HES has been shown to reduce amyloid‐β levels in LPS‐induced neuroinflammation via various pathways. These pathways encompass the control of inflammatory pathways, mitochondrial function, and direct contact with amyloid‐β peptides. The effects of HES are facilitated by its transformation into hesperetin within the body, which demonstrates neuroprotective properties by influencing critical signaling pathways and cellular mechanisms related to neuroinflammation and amyloid disease [83–86]. Moreover, CUR prevents amyloid aggregation and decreases neuroinflammation and these effects rely on TNF‐α receptor 2 activation [87, 88]. Regarding amyloid‐β, ISL reduces inflammatory cytokines and relieves oxidative stress via activating Nrf2 and decreasing NF‐κB [89]. Furthermore, ISL exhibits anti‐inflammatory properties by reducing TNF‐α through various mechanisms. It inhibits the NF‐κB pathway, thereby decreasing proinflammatory cytokine production. It further modulates MAPK pathways by suppressing JNK and p38 MAPK activation, and it directly interacts with myeloid differentiation protein‐2 to impede TNF‐alpha signaling. Additionally, ISL activates the Nrf2 pathway, enhancing antioxidant gene expression and reducing oxidative stress. It also inhibits the NOD‐, LRR‐, and pyrin domain‐containing protein 3 (NLRP3) inflammasome, interfering with proinflammatory cytokine maturation [28, 29, 90–92].

The intricate interplay affecting cognitive processes is further exemplified by the involvement of caspase‐3 and NO in memory formation in mice. The reduction of caspase‐3 exclusively impacts long‐term memory. Furthermore, the cleavage of tau protein by caspase‐3 in AD correlates with neurodegeneration and cognitive decline [93, 94]. Decreased NO production influences memory formation via various mechanisms. NO is vital for synaptic plasticity and memory; its dysregulation poses cognitive risks. LPS triggers neuroinflammation, raising inflammatory cytokines like TNF‐α and IL‐6, thus disrupting memory. Increased NO production from LPS can exacerbate oxidative stress and cognitive deficits. Modulating NO levels is crucial; inhibiting iNOS may alleviate memory deficits caused by inflammation. The complex role of NO suggests that while decreased levels can enhance memory by reducing neuroinflammation, complete inhibition could harm cognitive functions. Therefore, therapeutic approaches should seek a balance in NO levels to optimize cognitive outcomes amidst neuroinflammation [95–97]. Thereby, both molecules influence cognitive function and synaptic plasticity in different manners; the ratio of NO to caspase‐3 is crucial for cognitive function.

Secondary to inflammation, immune cells release large amounts of reactive oxygen species, which increase the oxidative stress and cause neuronal cell injury [75]. In our study, the tested compounds alleviated oxidative stress in cultured microglial cells and in mice hippocampi. In in vitro experiments, HES was capable of restoring GPx activity after LPS treatment. HES and CUR restored activity of GST compared to the LPS group. ISL and CUR enhanced SOD activity compared to the untreated LPS group. Likewise, in vivo, HES enhanced hippocampal GPx activity. Moreover, hippocampal GST and SOD activities were significantly augmented by treatment with HES, ISL, and CUR compared to LPS‐treated mice. In mice, HES displayed the highest stimulatory effect on antioxidant enzyme activity. Furthermore, HES, ISL, and CUR treatment decreased hippocampal MDA levels significantly compared to LPS‐treated mice. This could be partially attributed to the polyphenolic nature of the compounds and their chemical structure, as they can donate electrons or hydrogens to scavenge the free radicals as well as act as a chelating agent to ions, such as Fe^2+^, thereby preventing oxidation induced by hydroxyl radicals [98].

Targeting Nrf2 is a valuable therapeutic strategy for the treatment of neuroinflammation. A formulation of dimethyl fumarate, BG12, which targets the Nrf2 pathway, has been approved by the FDA for treatment of the relapsing type of multiple sclerosis [99]. The concentrations of Nrf2 protein in the nucleus and cytoplasm are vital for understanding the neuroinflammatory effects of natural compounds like HES, CUR, and ISL. In the current study, these compounds enhanced Nrf2 ratio levels, contributing to cellular defense against oxidative stress and inflammation. It has been reported that HES increases Nrf2 and its target HO‐1, reducing oxidative stress in LPS‐induced neuroinflammation. CUR restores Nrf2 levels in neurotoxic settings and boosts antioxidant enzymes, facilitating neuroprotection. ISL promotes Nrf2 translocation from cytoplasm to nucleus in traumatic brain injury models and suppresses NF‐κB signaling to diminish neuroinflammation [100–103]. Furthermore, the increase in the Nrf2 ratio in all treatments indicated that the treatments also act via the delocalization of the Nrf2, explaining the reduction in the NO in the BV‐2 cells and the decrease in the IL‐1β and oxidative stress in mice hippocampi [104].

Accumulating evidence implies that apoptosis is the main mechanism of cell death in chronic neurodegenerative diseases. Increased caspase activity is among the derangements found in the affected neurons in AD and ALS patients [105, 106]. This can be caused by elevated IL‐1β [107]. In our study, ISL showed the highest decrease in hippocampal caspase‐3 levels, followed by HES, implicating their anti‐apoptotic effect at the tested doses and the ability to alleviate LPS‐induced apoptosis in mice hippocampi. This was further confirmed through the decrease of caspase‐3 expression ratio in the brain cortex in all treatments compared to LPS through western blotting. CUR, HES, and ISL inactivate caspases to induce apoptosis through distinct mechanisms: CUR emphasizes p38‐mediated activation, HES predominantly affects intrinsic pathways as the JNK/Bax signaling pathway and gene expression, while ISL targets mitochondrial pathways and reactive oxygen species formation [108–110]. It has been reported that TNF‐α is an upstream marker that mediates apoptosis [111]. In the current study, all treatments were effective in decreasing the level of TNF‐α and hence inhibiting apoptosis that results in neurodegeneration.

In the present study, all treatments have demonstrated neuroprotective effects against LPS‐induced neuroinflammation in brain cortex models. This was further illustrated in the histopathological examination of the brain cortex. CUR crosses the blood–brain barrier, reducing inflammatory signaling via IL‐1β and COX‐2, and protecting against oxidative damage [112], while also inhibiting microglial activation and improving motor dysfunction [113, 114]. Moreover, CUR modulates the JNK/NF‐κB/Akt signaling pathway, reducing oxidative stress and ameliorating neuroinflammation and neuronal cell death [115]. ISL enhances antioxidant capacity, suppresses neuroinflammation, and regulates mitochondrial function to mitigate cognitive impairment; enhances the expression of NRF2‐responsive antioxidant genes; and suppresses NF‐κB‐responsive proinflammatory genes [69, 89–91]. HES shows considerable neuroprotective effects in neurodegenerative disorders. Its therapeutic potential stems from its antioxidant and anti‐inflammatory properties, enhancing endogenous defenses and reducing proinflammatory cytokines. HES modulates apoptotic pathways by decreasing caspase‐3 activity and protecting mitochondrial function. It also inhibits key enzymes involved in neurotransmitter breakdown, thus supporting cholinergic and dopaminergic systems. In disease models, it reduces amyloid‐β levels in AD [83, 116–120].

## 5. Conclusion

In conclusion, our study illustrates that ISL, HES, and CUR are neuroprotective agents that suppress oxidative stress, neuroinflammation, and reduce apoptosis and cognitive deficits secondary to LPS. This suggests their potential as therapeutics for neurodegenerative diseases. Limitations of this study include the difficulty of finding human subjects to further test the effects of the different treatments. So as a future recommendation, the treatments could be inserted in nanoparticle formulations to enhance their bioavailability and further be tested in human subjects.

**Nomenclature**
AD: Alzheimer’s disease
ALS: Amyotrophic lateral sclerosis
CNS: Central nervous system
CUR: Curcumin
GPx: Glutathione peroxidase
GST: Glutathione‐S‐transferase
HES: Hesperidin
HRP: Horseradish peroxidase
IL: Interleukin
ISL: Isoliquiritigenin
iNOS: Inducible nitric oxide synthase
LPS: Lipopolysaccharide
MWM: Morris water maze
NO: Nitric oxide
NLRP3: NOD‐, LRR‐ and pyrin domain‐containingprotein3
Nrf2: Nuclear factor erythroid 2‐related factor 2
OFT: Open field test
ORT: Object recognition test
PD: Parkinson’s disease
SOD: Superoxide dismutase
TNF‐α: Tumor necrosis factor‐alpha
TLR4: Toll‐like receptor 4

## Author Contributions

All authors shared in the study conception and design. Mona Elkhatieb, Rasha A. Radwan, Ahmed M. Hafez, Doaa Abou El‐ezz, Christine Adel Sedky, Nabila Hamdi, Sarah Atef Fahim, Amira Emad Abdelaziz, and Ulrike Breitinger performed material preparation, data collection, and analysis. Mona Elkhatieb and Doaa Abou El‐ezz wrote the first draft, and all authors commented on previous versions of the manuscript. Rasha A. Radwan wrote the final version with amendments.

## Funding

The authors affirm that they did not accept any grants, funding, or other assistance in order to prepare this manuscript.

## Disclosure

All authors have read and accepted the latest version of the manuscript.

## Ethics Statement

Institutional Review Board Statement: The investigation was performed in accordance with the Declaration of Helsinki, the guidelines of the Ethics Committee at MSA (approval number: PH1/EC1/2023PD), and the recommendations of the National Institutes of Health Guide for Care and Use of Laboratory Animals (Publication No. 8523, revised 1985). ARRIVE guidelines have been followed for all the animal experiments.

## Conflicts of Interest

The authors declare no conflicts of interest.

## Data Availability

All data generated or analyzed during this study are included within the article.

## References

[1] Hwang S.-L. , Shih P.-H. , and Yen G.-C. , Citrus Flavonoids and Effects in Dementia and Age-Related Cognitive Decline, Diet and Nutrition in Dementia and Cognitive Decline. (2015) Elsevier, 869–878, 10.1016/B978-0-12-407824-6.00080-X, 2-s2.0-84943273850.

[2] Adamu A. , Li S. , Gao F. , and Xue G. , The Role of Neuroinflammation in Neurodegenerative Diseases: Current Understanding and Future Therapeutic Targets, Frontiers in Aging Neuroscience. (2024) 16, 10.3389/fnagi.2024.1347987. PMC11045904 38681666

[3] Alemán-Villa K. M. , Armienta-Rojas D. A. , Camberos-Barraza J. et al., Neuroinflammation Across the Spectrum of Neurodegenerative Diseases: Mechanisms and Therapeutic Frontiers, Neuroimmunomodulation. (2025) 32, 278–305, 10.1159/000548021. 40931498

[4] Mattson M. P. , Apoptosis in Neurodegenerative Disorders, Nature Reviews Molecular Cell Biology. (2000) 1, no. 2, 120–130, 10.1038/35040009. 11253364

[5] Cook A. L. , Vitale A. M. , Ravishankar S. et al., NRF2 Activation Restores Disease Related Metabolic Deficiencies in Olfactory Neurosphere-Derived Cells from Patients with Sporadic Parkinson’s Disease, PLoS One. (2011) 6, no. 7, 10.1371/journal.pone.0021907, 2-s2.0-79959776301. PMC3128624 21747966

[6] Ramsey C. P. , Glass C. A. , Montgomery M. B. et al., Expression of Nrf2 in Neurodegenerative Diseases, Journal of Neuropathology & Experimental Neurology. (2007) 66, no. 1, 75–85, 10.1097/nen.0b013e31802d6da9, 2-s2.0-33846073880. 17204939 PMC2253896

[7] Vargas M. R. , Johnson D. A. , Sirkis D. W. , Messing A. , and Johnson J. A. , Nrf2 Activation in Astrocytes Protects Against Neurodegeneration in Mouse Models of Familial Amyotrophic Lateral Sclerosis, Journal of Neuroscience. (2008) 28, no. 50, 13574–13581, 10.1523/JNEUROSCI.4099-08.2008, 2-s2.0-58149379610. 19074031 PMC2866507

[8] Kim K. H. , Lyu J. H. , Koo S. T. et al., MyD88 is a Mediator for the Activation of Nrf2, Biochemical and Biophysical Research Communications. (2011) 404, no. 1, 46–51, 10.1016/j.bbrc.2010.11.051, 2-s2.0-78650860386. 21094136

[9] Wu Z. , Huang Y. , Hu W. et al., Lipopolysaccharide-Induced Inflammation Increases Nitric Oxide Production in Taste Buds, Brain, Behavior, and Immunity. (2022) 103, 145–153, 10.1016/j.bbi.2022.04.016. 35447301 PMC10353706

[10] Chhor V. , Le Charpentier T. , Lebon S. et al., Characterization of Phenotype Markers and Neuronotoxic Potential of Polarised Primary Microglia in Vitro, Brain, Behavior, and Immunity. (2013) 32, 70–85, 10.1016/j.bbi.2013.02.005, 2-s2.0-84891373720. 23454862 PMC3694309

[11] Henn A. , The Suitability of BV2 Cells as Alternative Model System for Primary Microglia Cultures or for Animal Experiments Examining Brain Inflammation, ALTEX. (2009) 83–94, 10.14573/altex.2009.2.83. 19565166

[12] Chen Y. , Peng F. , Xing Z. , Chen J. , Peng C. , and Li D. , Beneficial Effects of Natural Flavonoids on Neuroinflammation, Frontiers in Immunology. (2022) 13, 10.3389/fimmu.2022.1006434. PMC9638012 36353622

[13] Subramanian P. , Anandan R. , Jayapalan J. J. , and Hashim O. H. , Hesperidin Protects gentamicin-induced Nephrotoxicity via Nrf2/HO-1 Signaling and Inhibits Inflammation Mediated by NF-κB in Rats, Journal of Functional Foods. (2015) 13, 89–99, 10.1016/j.jff.2014.12.035, 2-s2.0-84922569213.

[14] Gong H. , Zhang B. , Yan M. et al., A Protective Mechanism of Licorice (Glycyrrhiza Uralensis): Isoliquiritigenin Stimulates Detoxification System via Nrf2 Activation, Journal of Ethnopharmacology. (2015) 162, 134–139, 10.1016/j.jep.2014.12.043, 2-s2.0-84921419288. 25557030

[15] Abou El-ezz D. , Maher A. , Sallam N. , El-brairy A. , and Kenawy S. , Trans-Cinnamaldehyde Modulates Hippocampal Nrf2 Factor and Inhibits Amyloid Beta Aggregation in LPS-Induced Neuroinflammation Mouse Model, Neurochemical Research. (2018) 43, no. 12, 2333–2342, 10.1007/s11064-018-2656-y, 2-s2.0-85055050872. 30302613

[16] Atoki A. V. , Aja P. M. , Shinkafi T. S. , Ondari E. N. , and Awuchi C. G. , Hesperidin Plays Beneficial Roles in Disorders Associated With the Central Nervous System: A Review, International Journal of Food Properties. (2023) 26, no. 1, 1867–1884, 10.1080/10942912.2023.2236327.

[17] Balkrishna A. , Sinha S. , Kumar A. et al., Sepsis-Mediated Renal Dysfunction: Pathophysiology, Biomarkers and Role of Phytoconstituents in its Management, Biomedicine & Pharmacotherapy. (2023) 165, 10.1016/j.biopha.2023.115183. 37487442

[18] Bai Y. , Zhou J. , Zhu H. et al., Isoliquiritigenin Inhibits Microglia-Mediated Neuroinflammation in Models of Parkinson’s Disease via JNK/AKT/NFκB Signaling Pathway, Phytotherapy Research. (2023) 37, no. 3, 848–859, 10.1002/ptr.7665. 36484427

[19] Azzini E. , Peña‐Corona S. I. , Hernández-Parra H. et al., Neuroprotective and Anti‐Inflammatory Effects of Curcumin in Alzheimer’s Disease: Targeting Neuroinflammation Strategies, Phytotherapy Research. (2024) 38, no. 6, 3169–3189, 10.1002/ptr.8200. 38616356

[20] Chen B. , Shi Q. , Nie C. et al., Curcumin Alleviates Oxidative Stress, Neuroinflammation, and PromotesBehavioral Recovery After Traumatic Brain Injury, CNR. (2023) 20, no. 1, 43–53, 10.2174/1567202620666230303144323. 36872351

[21] Chen Y.-C. , Kuo T.-C. , Lin-Shiau S.-Y. , and Lin J.-K. , Induction ofHSP70 Gene Expression by Modulation of Ca+2 Ion and Cellular p53 Protein by Curcumin in Colorectal Carcinoma Cells, Molecular Carcinogenesis. (1996) 17, no. 4, 224–234. 8989916 10.1002/(SICI)1098-2744(199612)17:4<224::AID-MC6>3.0.CO;2-D

[22] Zafar A. , Lahori D. , Namit A. F. et al., Curcumin in Inflammatory Complications: Therapeutic Applications and Clinical Evidence, Indian Journal of Management Science. (2025) 26, no. 19, 10.3390/ijms26199366. PMC12524702 41096639

[23] El-Saadony M. T. , Saad A. M. , Mohammed D. M. et al., Curcumin, an Active Component of Turmeric: Biological Activities, Nutritional Aspects, Immunological, Bioavailability, and Human Health Benefits-a Comprehensive Review, Frontiers in Immunology. (2025) 16, 10.3389/fimmu.2025.1603018. PMC12408333 40918117

[24] Pal R. S. , Pal Y. , Punniyakotti S. , Katiyar D. , and Kumari P. , Hesperidin: Diversified Prospects of Naturally Occurring Bioflavonoid, NPJ. (2024) 14, no. 3, 10.2174/2210315514666230816141802.

[25] Pyrzynska K. , Hesperidin: A Review on Extraction Methods, Stability and Biological Activities, Nutrients. (2022) 14, no. 12, 10.3390/nu14122387. PMC9227685 35745117

[26] Chen Z. , Ding W. , Yang X. , Lu T. , and Liu Y. , Isoliquiritigenin, a Potential Therapeutic Agent for Treatment of Inflammation-Associated Diseases, Journal of Ethnopharmacology. (2024) 318, 10.1016/j.jep.2023.117059. 37604329

[27] Peng F. , Du Q. , Peng C. et al., A Review: The Pharmacology of Isoliquiritigenin, Phytotherapy Research. (2015) 29, no. 7, 969–977, 10.1002/ptr.5348, 2-s2.0-84936866336. 25907962

[28] Zhu X. , Liu J. , Huang S. et al., Neuroprotective Effects of Isoliquiritigenin Against Cognitive Impairment via Suppression of Synaptic Dysfunction, Neuronal Injury, and Neuroinflammation in Rats With Kainic Acid-Induced Seizures, International Immunopharmacology. (2019) 72, 358–366, 10.1016/j.intimp.2019.04.028, 2-s2.0-85064598625. 31030091

[29] Lee D. G. , Nam B. R. , Huh J.-W. , and Lee D.-S. , Isoliquiritigenin Reduces LPS-Induced Inflammation by Preventing Mitochondrial Fission in BV-2 Microglial Cells, Inflammation. (2021) 44, no. 2, 714–724, 10.1007/s10753-020-01370-2. 33150538

[30] Jin C. , Lee J. , Park C. , Choi Y. H. , and Kim G. , Curcumin Attenuates the Release of Pro-Inflammatory Cytokines in Lipopolysaccharide-Stimulated BV2 Microglia, Acta Pharmacologica Sinica. (2007) 28, no. 10, 1645–1651, 10.1111/j.1745-7254.2007.00651.x, 2-s2.0-34848821886. 17883952

[31] Foresti R. , Bains S. K. , Pitchumony T. S. et al., Small Molecule Activators of the Nrf2-HO-1 Antioxidant Axis Modulate Heme Metabolism and Inflammation in BV2 Microglia Cells, Pharmacological Research. (2013) 76, 132–148, 10.1016/j.phrs.2013.07.010, 2-s2.0-84883412049. 23942037

[32] Jung K. K. , Lee H. S. , Cho J. Y. et al., Inhibitory Effect of Curcumin on Nitric Oxide Production From Lipopolysaccharide-Activated Primary Microglia, Life Sciences. (2006) 79, no. 21, 2022–2031, 10.1016/j.lfs.2006.06.048, 2-s2.0-33749533244. 16934299

[33] Filannino F. M. , Soleti R. , Ruggiero M. et al., Chrysin-Loaded Extracellular Vesicles Attenuate LPS-Induced Neuroinflammation in BV2 Microglial Cells in Vitro: A Novel Neuroprotective Strategy, Molecules. (2025) 30, no. 15, 10.3390/molecules30153131. PMC12348394 40807306

[34] Lee S.-G. and Kang H. , Effect of Ethanol Extracts from Defatted *Perilla frutescens* on LPS-Induced Inflammation in Mouse BV2 Microglial Cells, BSL. (2018) 24, no. 4, 398–404, 10.15616/BSL.2018.24.4.398.

[35] Dang T. K. , Hong S.-M. , Dao V. T. et al., Anti-Neuroinflammatory Effects of Alkaloid-Enriched Extract From *Huperzia serrata* on Lipopolysaccharide-Stimulated BV-2 Microglial Cells, Pharmaceutical Biology. (2023) 61, no. 1, 135–143, 10.1080/13880209.2022.2159450. 36617895 PMC9833413

[36] Cahoon D. S. , Fisher D. R. , Lamon-Fava S. , Wu D. , Zheng T. , and Shukitt-Hale B. , Blueberry Treatment Administered Before and/or After Lipopolysaccharide Stimulation Attenuates Inflammation and Oxidative Stress in Rat Microglial Cells, Nutritional Neuroscience. (2023) 26, no. 2, 127–137, 10.1080/1028415X.2021.2020404. 36692990

[37] Alaufi O. M. , Noorwali A. , Zahran F. , Al-Abd A. M. , and Al-Attas S. , Cytotoxicity of Thymoquinone Alone or in Combination With Cisplatin (CDDP) Against Oral Squamous Cell Carcinoma in Vitro, Scientific Reports. (2017) 7, no. 1, 10.1038/s41598-017-13357-5, 2-s2.0-85031670847. PMC5640598 29030590

[38] Maher A. , El-Sayed N. S.-E. , Breitinger H.-G. , and Gad M. Z. , Overexpression of NMDAR2B in an Inflammatory Model of Alzheimer’s Disease: Modulation by NOS Inhibitors, Brain Research Bulletin. (2014) 109, 109–116, 10.1016/j.brainresbull.2014.10.007, 2-s2.0-84910048025. 25454121

[39] Wang C. , Tsai Y. , Hsieh Y. , Lin R. , and Lin C. , The Aqueous Extract from *Toona sinensis* Leaves Inhibits Microglia-Mediated Neuroinflammation, The Kaohsiung Journal of Medical Sciences. (2014) 30, no. 2, 73–81, 10.1016/j.kjms.2013.09.012, 2-s2.0-84892787182. 24444536 PMC7118447

[40] Bradford M. M. , A Rapid and Sensitive Method for the Quantitation of Microgram Quantities of Protein Utilizing the Principle of protein-dye Binding, Analytical Biochemistry. (1976) 72, no. 1-2, 248–254, 10.1016/0003-2697(76)90527-3, 2-s2.0-0017184389. 942051

[41] Peskin A. V. and Winterbourn C. C. , Assay of Superoxide Dismutase Activity in a Plate Assay Using WST-1, Free Radical Biology and Medicine. (2017) 103, 188–191, 10.1016/j.freeradbiomed.2016.12.033, 2-s2.0-85007358171. 28017897

[42] Weydert C. J. and Cullen J. J. , Measurement of Superoxide Dismutase, Catalase and Glutathione Peroxidase in Cultured Cells and Tissue, Nature Protocols. (2010) 5, no. 1, 51–66, 10.1038/nprot.2009.197, 2-s2.0-75149164344. 20057381 PMC2830880

[43] Habig W. H. , Pabst M. J. , and Jakoby W. B. , Glutathione S-transferases. the First Enzymatic Step in Mercapturic Acid Formation, Journal of Biological Chemistry. (1974) 249, no. 22, 7130–7139, 10.1016/s0021-9258(19)42083-8. 4436300

[44] Pan J. , Li H. , Ma J.-F. et al., Curcumin Inhibition of JNKs Prevents Dopaminergic Neuronal Loss in a Mouse Model of Parkinson’s Disease through Suppressing Mitochondria Dysfunction, Translational Neurodegeneration. (2012) 1, 10.1186/2047-9158-1-16, 2-s2.0-84874663567. PMC3514118 23210631

[45] Wang Z. , Wang N. , Han S. et al., Dietary Compound Isoliquiritigenin Inhibits Breast Cancer Neoangiogenesis via VEGF/VEGFR-2 Signaling Pathway, PLoS One. (2013) 8, no. 7, 10.1371/journal.pone.0068566, 2-s2.0-84879814057. PMC3702614 23861918

[46] Javed H. , Vaibhav K. , Ahmed M. E. et al., Effect of Hesperidin on Neurobehavioral, Neuroinflammation, Oxidative Stress and Lipid Alteration in Intracerebroventricular Streptozotocin Induced Cognitive Impairment in Mice, Journal of the Neurological Sciences. (2015) 348, no. 1-2, 51–59, 10.1016/j.jns.2014.10.044, 2-s2.0-84920987667. 25434716

[47] Atya H. B. , Sharaf N. M. , Abdelghany R. M. , El-Helaly S. N. , and Taha H. , Autophagy and Exosomes; Inter-Connected Maestros in Alzheimer’s Disease, Inflammopharmacology. (2024) 32, no. 3, 2061–2073, 10.1007/s10787-024-01466-3. 38564092 PMC11136856

[48] Sorial M. E. S. , Abdelghany R. M. , and El S. N. S. E. D. , Modulation of the Cognitive Impairment Associated with Alzheimer’s Disease by Valproic Acid: Possible Drug Repurposing, Inflammopharmacology. (2025) 33, no. 4, 2083–2094, 10.1007/s10787-025-01695-0. 40108007 PMC11991970

[49] Sato K. , Miyakawa K. , Takeya M. et al., Immunohistochemical Expression of Inducible Nitric Oxide Synthase (iNOS) in Reversible Endotoxic Shock Studied by a Novel Monoclonal Antibody Against Rat iNOS, Journal of Leukocyte Biology. (1995) 57, no. 1, 36–44, 10.1002/jlb.57.1.36. 7530282

[50] Kim S.-H. , Han J. , Seog D.-H. et al., Antidepressant Effect of Chaihu-Shugan-San Extract and Its Constituents in Rat Models of Depression, Life Sciences. (2005) 76, no. 11, 1297–1306, 10.1016/j.lfs.2004.10.022, 2-s2.0-11844266513. 15642599

[51] Ennaceur A. , One-Trial Object Recognition in Rats and Mice: Methodological and Theoretical Issues, Behavioural Brain Research. (2010) 215, no. 2, 244–254, 10.1016/j.bbr.2009.12.036, 2-s2.0-77954317073. 20060020

[52] Vorhees C. V. and Williams M. T. , Morris Water Maze: Procedures for Assessing Spatial and Related Forms of Learning and Memory, Nature Protocols. (2006) 1, no. 2, 848–858, 10.1038/nprot.2006.116, 2-s2.0-33846461062. 17406317 PMC2895266

[53] Emad B. , WalyEldeen A. A. , Hassan H. et al., Yttrium Oxide Nanoparticles Induce Cytotoxicity, Genotoxicity, Apoptosis, and Ferroptosis in the Human Triple-Negative Breast Cancer MDA-MB-231 Cells, BMC Cancer. (2023) 23, no. 1, 10.1186/s12885-023-11649-w. PMC10680179 38012585

[54] Suvarna S. K. , Layton C. , and Bancroft J. D. , Bancroft’s Theory and Practice of Histological Techniques, 2019, 8th edition, Elsevier, Amsterdam.

[55] Fu Y. , Yang P. , Zhao Y. et al., *Trans* ‐Cinnamaldehyde Inhibits Microglial Activation and Improves Neuronal Survival Against Neuroinflammation in BV2 Microglial Cells with Lipopolysaccharide Stimulation, Evidence-Based Complementary and Alternative Medicine. (2017) 2017, no. 1, 10.1155/2017/4730878, 2-s2.0-85042234907. PMC5671715 29234401

[56] Ho S.-C. , Chang K.-S. , and Chang P.-W. , Inhibition of Neuroinflammation by Cinnamon and Its Main Components, Food Chemistry. (2013) 138, no. 4, 2275–2282, 10.1016/j.foodchem.2012.12.020, 2-s2.0-84875159906. 23497886

[57] Sakata K. , Hirose Y. , Qiao Z. , Tanaka T. , and Mori H. , Inhibition of Inducible Isoforms of Cyclooxygenase and Nitric Oxide Synthase by Flavonoid Hesperidin in Mouse Macrophage Cell Line, Cancer Letters. (2003) 199, no. 2, 139–145, 10.1016/S0304-3835(03)00386-0, 2-s2.0-0142060124. 12969786

[58] Gallorini M. , Rapino M. , Schweikl H. , Cataldi A. , Amoroso R. , and Maccallini C. , Selective Inhibitors of the Inducible Nitric Oxide Synthase as Modulators of Cell Responses in LPS-Stimulated Human Monocytes, Molecules. (2021) 26, no. 15, 10.3390/molecules26154419. PMC8348305 34361571

[59] Nagai Y. , Watanabe Y. , Honda H. , and Takatsu K. , Sakagami H. , Isoliquiritigenin: a Unique Component that Attenuates Adipose Tissue Inflammation and Fibrosis by Targeting the Innate Immune Sensors, Biological Activities and Action Mechanisms of Licorice Ingredients, 2017, InTech, 10.5772/66727.

[60] Lee J.-W. , Bae C. J. , Choi Y.-J. et al., 3,4,5-Trihydroxycinnamic Acid Inhibits LPS-Induced iNOS Expression by Suppressing NF-κB Activation in BV2 Microglial Cells, Korean Journal of Physiology and Pharmacology. (2012) 16, no. 2, 10.4196/kjpp.2012.16.2.107, 2-s2.0-84861016739. PMC3339285 22563255

[61] Cannataro R. , Fazio A. , La Torre C. , Caroleo M. C. , and Cione E. , Polyphenols in the Mediterranean Diet: from Dietary Sources to microRNA Modulation, Antioxidants. (2021) 10, no. 2, 10.3390/antiox10020328. PMC7926722 33672251

[62] Kwon Y. , Estimation of Curcumin Intake in Korea Based on the Korea National Health and Nutrition Examination Survey (2008-2012), Nutrition Research and Practice. (2014) 8, no. 5, 10.4162/nrp.2014.8.5.589, 2-s2.0-84908079579. PMC4198974 25324941

[63] Omar H. R. , Komarova I. , El-Ghonemi M. et al., Licorice Abuse: Time to Send a Warning Message, Therapeutic Advances in Endocrinology. (2012) 3, no. 4, 125–138, 10.1177/2042018812454322, 2-s2.0-84864663042. PMC3498851 23185686

[64] Lim T. K. , Glycyrrhiza glabra, Edible Medicinal and Non-Medicinal Plants. (2016) Springer Netherlands, Dordrecht, 354–457, 10.1007/978-94-017-7276-1_18.

[65] Correa F. , Ljunggren E. , Patil J. et al., Time-Dependent Effects of Systemic Lipopolysaccharide Injection on Regulators of Antioxidant Defence Nrf2 and PGC-1α in the Neonatal Rat Brain, Neuroimmunomodulation. (2013) 20, no. 4, 185–193, 10.1159/000347161, 2-s2.0-84876807588. 23635713 PMC4096332

[66] Lund S. , Christensen K. V. , Hedtjärn M. et al., The Dynamics of the LPS Triggered Inflammatory Response of Murine Microglia Under Different Culture and in Vivo Conditions, Journal of Neuroimmunology. (2006) 180, no. 1-2, 71–87, 10.1016/j.jneuroim.2006.07.007, 2-s2.0-33750627921. 16996144

[67] Li C. , Zug C. , Qu H. , Schluesener H. , and Zhang Z. , Hesperidin Ameliorates Behavioral Impairments and Neuropathology of Transgenic APP/PS1 Mice, Behavioural Brain Research. (2015) 281, 32–42, 10.1016/j.bbr.2014.12.012, 2-s2.0-84920876906. 25510196

[68] Yue Y.-K. , Mo B. , Zhao J. et al., Neuroprotective Effect of Curcumin Against Oxidative Damage in BV-2 Microglia and High Intraocular Pressure Animal Model, Journal of Ocular Pharmacology and Therapeutics. (2014) 30, no. 8, 657–664, 10.1089/jop.2014.0022, 2-s2.0-84912026274. 24963995

[69] Zhu X. , Liu J. , Chen S. et al., Isoliquiritigenin Attenuates lipopolysaccharide-induced Cognitive Impairment Through Antioxidant and Anti-inflammatory Activity, BMC Neuroscience. (2019) 20, no. 1, 10.1186/s12868-019-0520-x, 2-s2.0-85070256879. PMC6685153 31387531

[70] Khan H. , Naseem T. , Kaushik P. et al., Decoding Paradoxical Links of Cytokine Markers in Cognition: Cross Talk Between Physiology, Inflammaging, and Alzheimer’s disease-Related Cognitive Decline, Ageing Research Reviews. (2024) 101, 10.1016/j.arr.2024.102535. 39374831

[71] Gullo F. , Ceriani M. , D’Aloia A. et al., Plant Polyphenols and Exendin-4 Prevent Hyperactivity and TNF-α Release in LPS-Treated in Vitro Neuron/Astrocyte/Microglial Networks, Frontiers in Neuroscience. (2017) 11, 10.3389/fnins.2017.00500, 2-s2.0-85028915787. PMC5592223 28932183

[72] Hazen J. , Vistnes M. , Barca M. L. et al., The Association Between Circulating Inflammatory Markers and the Progression of Alzheimer Disease in Norwegian Memory Clinic Patients With Mild Cognitive Impairment or Dementia, Alzheimer Disease and Associated Disorders. (2020) 34, no. 1, 47–53, 10.1097/WAD.0000000000000342, 2-s2.0-85071260935. 31414991

[73] Karima S. , Mahdavi M. , Rajaei S. et al., Plasma Cytokines Profile in Subjects with Alzheimerâ€™S Disease: Interleukin 1 Alpha as a Candidate for Target Therapy, Galen Medical Journal. (2021) 10, 10.31661/gmj.v10i.1974. PMC9007609 35434157

[74] Pacoova Dal Maschio V. , Roveta F. , Bonino L. , Boschi S. , Rainero I. , and Rubino E. , The Role of Blood-Based Biomarkers in Transforming Alzheimer’s Disease Research and Clinical Management: A Review, Indian Journal of Management Science. (2025) 26, no. 17, 10.3390/ijms26178564. PMC12428803 40943483

[75] Chatterjee S. , Oxidative Stress, Inflammation, and Disease, Oxidative Stress and Biomaterials. (2016) Elsevier, 35–58, 10.1016/B978-0-12-803269-5.00002-4, 2-s2.0-85018844329.

[76] Sarto J. , Ruiz-García R. , Guillén N. et al., Diagnostic Performance and Clinical Applicability of Blood-Based Biomarkers in a Prospective Memory Clinic Cohort, Neurology. (2023) 100, 10.1212/WNL.0000000000201597. PMC9984216 36450604

[77] Li M. , Shao H. , Zhang X. , and Qin B. , Hesperidin Alleviates Lipopolysaccharide-Induced Neuroinflammation in Mice by Promoting the miRNA-132 Pathway, Inflammation. (2016) 39, no. 5, 1681–1689, 10.1007/s10753-016-0402-7, 2-s2.0-84976868777. 27378528

[78] Javan N. , Khadem Ansari M. H. , Dadashpour M. et al., Synergistic Antiproliferative Effects of Co-nanoencapsulated Curcumin and Chrysin on MDA-MB-231 Breast Cancer Cells Through Upregulating miR-132 and miR-502c, Nutrition and Cancer. (2019) 71, no. 7, 1201–1213, 10.1080/01635581.2019.1599968, 2-s2.0-85063962616. 30955355

[79] Kong H. , Yin F. , He F. et al., The Effect of miR-132, miR-146a, and miR-155 on MRP8/TLR4-Induced Astrocyte-Related Inflammation, Journal of Molecular Neuroscience. (2015) 57, no. 1, 28–37, 10.1007/s12031-015-0574-x, 2-s2.0-84939566508. 25957996

[80] Kashif H. , Shah D. , and Sukumari-Ramesh S. , Dysregulation of microRNA and Intracerebral Hemorrhage: Roles in Neuroinflammation, Indian Journal of Management Science. (2021) 22, no. 15, 10.3390/ijms22158115. PMC8347974 34360881

[81] Wang Y. , Veremeyko T. , Wong A. H.-K. et al., Downregulation of miR-132/212 Impairs S-nitrosylation Balance and Induces Tau Phosphorylation in Alzheimer’s Disease, Neurobiology of Aging. (2017) 51, 156–166, 10.1016/j.neurobiolaging.2016.12.015, 2-s2.0-85009471312. 28089352

[82] Frankola K A. , Greig N H. , Luo W. , and Tweedie D. , Targeting TNF-Alpha to Elucidate and Ameliorate Neuroinflammation in Neurodegenerative Diseases, CNSNDDT. (2011) 10, 391–403, 10.2174/187152711794653751. PMC4663975 21288189

[83] Ikram M. , Muhammad T. , Rehman S. U. et al., Hesperetin Confers Neuroprotection by Regulating Nrf2/TLR4/NF-κB Signaling in an Aβ Mouse Model, Molecular Neurobiology. (2019) 56, no. 9, 6293–6309, 10.1007/s12035-019-1512-7, 2-s2.0-85061433799. 30756299

[84] Jafni S. , Sathya S. , Arunkumar M. et al., Hesperidin Methyl Chalcone Reduces Extracellular Aβ(25-35) Peptide Aggregation and Fibrillation and Also Protects Neuro 2a Cells From Aβ(25-35) Induced Neuronal Dysfunction, Bioorganic & Medicinal Chemistry. (2023) 96, 10.1016/j.bmc.2023.117536. 38016411

[85] Wang D.-M. , Li S.-Q. , Zhu X.-Y. , Wang Y. , Wu W.-L. , and Zhang X.-J. , Protective Effects of Hesperidin Against Amyloid-β (Aβ) Induced Neurotoxicity Through the Voltage Dependent Anion Channel 1 (VDAC1)-Mediated Mitochondrial Apoptotic Pathway in PC12 Cells, Neurochemical Research. (2013) 38, no. 5, 1034–1044, 10.1007/s11064-013-1013-4, 2-s2.0-84876187514. 23475456

[86] Muhammad T. , Ikram M. , Ullah R. , Rehman S. , and Kim M. , Hesperetin, a Citrus Flavonoid, Attenuates LPS-Induced Neuroinflammation, Apoptosis and Memory Impairments by Modulating TLR4/NF-κB Signaling, Nutrients. (2019) 11, no. 3, 10.3390/nu11030648, 2-s2.0-85063269063. PMC6471991 30884890

[87] Baj T. and Seth R. , Role of Curcumin in Regulation of TNF-α Mediated Brain Inflammatory Responses, IAD. (2018) 12, no. 1, 69–77, 10.2174/1872213X12666180703163824, 2-s2.0-85052330191. 29972106

[88] He W. , Yuan K. , Ji B. , Han Y. , and Li J. , Protective Effects of Curcumin Against Neuroinflammation Induced by Aβ25-35 in Primary Rat Microglia: Modulation of High-Mobility Group Box 1, Toll-like Receptor 4 and Receptor for Advanced Glycation End Products Expression, Annals of Translational Medicine. (2020) 8, 10.21037/atm.2019.12.147. PMC7048970 32175381

[89] Fu Y. and Jia J. , Isoliquiritigenin Confers Neuroprotection and Alleviates Amyloid-β42-Induced Neuroinflammation in Microglia by Regulating the Nrf2/NF-κB Signaling, Frontiers in Neuroscience. (2021) 15, 10.3389/fnins.2021.638772. PMC7904903 33642990

[90] Jin X. Y. , Sohn D. H. , and Lee S. H. , Isoliquiritigenin Suppresses Tumor Necrosis Factor-α-Induced Inflammation via Peroxisome Proliferator-Activated Receptor-γ in Intestinal Epithelial Cells, Archives of Pharmacal Research. (2016) 39, no. 10, 1465–1471, 10.1007/s12272-016-0805-x, 2-s2.0-84982306232. 27539609

[91] Liu Q. , Lv H. , Wen Z. , Ci X. , and Peng L. , Isoliquiritigenin Activates Nuclear Factor Erythroid-2 Related Factor 2 to Suppress the NOD-Like Receptor Protein 3 Inflammasome and Inhibits the NF-κB Pathway in Macrophages and in Acute Lung Injury, Frontiers in Immunology. (2017) 8, 10.3389/fimmu.2017.01518, 2-s2.0-85034116644. PMC5677786 29163554

[92] Yang L. , Nie H. , Du Y. , Liu X. , Cai B. , and Li J. , Isoliquiritigenin Exhibits Anti-Inflammatory Responses in Acute Lung Injury by Covalently Binding to the Myeloid Differentiation Protein-2 Domain, Phytotherapy Research. (2025) 39, no. 2, 922–937, 10.1002/ptr.8411. 39697044

[93] Płóciennik A. , Prendecki M. , Zuba E. , Siudzinski M. , and Dorszewska J. , Activated Caspase-3 and Neurodegeneration and Synaptic Plasticity in Alzheimer’s Disease, AAD. (2015) no. 04, 63–77, 10.4236/aad.2015.43007.

[94] High Serum caspase-3 Level Confers Poor Prognosis After TBI, Nature Reviews Neurology. (2015) 11, 10.1038/nrneurol.2015.211.

[95] Yamada K. , Role of Nitric Oxide in Learning and Memory Processes, Folia Pharmacologica Japonica. (1998) 111, no. 2, 87–96, 10.1254/fpj.111.87, 2-s2.0-0031916163. 9558647

[96] Wang X. , Wang H. , and Li H. , Inhibition of iNOS Ameliorates Traumatic Stress-Induced Deficits in Synaptic Plasticity and Memory, Psychiatry Research. (2018) 268, 413–418, 10.1016/j.psychres.2018.08.028, 2-s2.0-85051628861. 30125872

[97] Öz M. and Erdal H. , A TNF-α Inhibitor Abolishes sepsis-induced Cognitive Impairment in Mice by Modulating Acetylcholine and Nitric Oxide Homeostasis, BDNF Release, and Neuroinflammation, Behavioural Brain Research. (2024) 466, 10.1016/j.bbr.2024.114995. 38599251

[98] Tsao R. , Chemistry and Biochemistry of Dietary Polyphenols, Nutrients. (2010) 2, no. 12, 1231–1246, 10.3390/nu2121231, 2-s2.0-79952066487. 22254006 PMC3257627

[99] Linker R. A. , Lee D.-H. , Ryan S. et al., Fumaric Acid Esters Exert Neuroprotective Effects in Neuroinflammation via Activation of the Nrf2 Antioxidant Pathway, Brain. (2011) 134, no. 3, 678–692, 10.1093/brain/awq386, 2-s2.0-79952136166. 21354971

[100] Zhang M. , Huang L.-L. , Teng C.-H. et al., Isoliquiritigenin Provides Protection and Attenuates Oxidative Stress-Induced Injuries via the Nrf2-ARE Signaling Pathway After Traumatic Brain Injury, Neurochemical Research. (2018) 43, no. 12, 2435–2445, 10.1007/s11064-018-2671-z, 2-s2.0-85056768161. 30446968

[101] Navarro E. and Esteras N. , Multitarget Effects of Nrf2 Signalling in the Brain: Common and Specific Functions in Different Cell Types, Antioxidants. (2024) 13, no. 12, 10.3390/antiox13121502. PMC11673142 39765831

[102] Lan X. , Wang Q. , Liu Y. et al., Isoliquiritigenin Alleviates Cerebral Ischemia-Reperfusion Injury by Reducing Oxidative Stress and Ameliorating Mitochondrial Dysfunction via Activating the Nrf2 Pathway, Redox Biology. (2024) 77, 10.1016/j.redox.2024.103406. PMC11546133 39454290

[103] Sidiropoulou G. A. , Metaxas A. , and Kourti M. , Natural Antioxidants That Act Against Alzheimer’s Disease Through Modulation of the NRF2 Pathway: A Focus on their Molecular Mechanisms of Action, Frontiers in Endocrinology. (2023) 14, 10.3389/fendo.2023.1217730. PMC10351420 37465125

[104] González-Carnicero Z. , Hernanz R. , Martínez-Casales M. , Barrús M. T. , Martín Á. , and Alonso M. J. , Regulation by Nrf2 of IL-1β-induced Inflammatory and Oxidative Response in VSMC and Its Relationship With TLR4, Frontiers in Pharmacology. (2023) 14, 10.3389/fphar.2023.1058488. PMC10018188 36937865

[105] Martin L. J. , Neuronal Death in Amyotrophic Lateral Sclerosis Is Apoptosis: Possible Contribution of a Programmed Cell Death Mechanism, Journal of Neuropathology and Experimental Neurology. (1999) 58, no. 5, 459–471, 10.1097/00005072-199905000-00005, 2-s2.0-0032896327. 10331434

[106] Masliah E. , Mallory M. , Alford M. , Tanaka S. , and Hansen L. A. , Caspase Dependent DNA Fragmentation Might Be Associated With Excitotoxicity in Alzheimer Disease, Journal of Neuropathology and Experimental Neurology. (1998) 57, no. 11, 1041–1052, 10.1097/00005072-199811000-00007, 2-s2.0-0031760611. 9825941

[107] Nesic O. , Xu G.-Y. , McAdoo D. , Westlund High K. , Hulsebosch C. , and Perez-Polo R. , IL-1 Receptor Antagonist Prevents Apoptosis and Caspase-3 Activation After Spinal Cord Injury, Journal of Neurotrauma. (2001) 18, no. 9, 947–956, 10.1089/089771501750451857, 2-s2.0-0034856386. 11565605

[108] Novinbahador T. , Araj-Khodaei M. , and Mahdavi M. , Evidence for Hesperidin as an Effective Factor in Initiating the Intrinsic Pathway of Apoptosis in KG1a Leukemia Cells, International Journal of Toxicology. (2023) 42, no. 2, 165–171, 10.1177/10915818221146468. 36534417

[109] Hsueh K. , Ju P. , Hsieh Y. , Su S. , Yeh C. , and Lin C. , HO-3867, a Curcumin Analog, Elicits Cell Apoptosis and p38-Mediated Caspase Activation in Hepatocellular Carcinoma, Environmental Toxicology. (2024) 39, no. 2, 794–802, 10.1002/tox.23977. 37782689

[110] Hwang M. , Kwon M. , and Kim B. , Isoliquiritigenin Induces Apoptosis Through Caspases and Reactive Oxygen Species Signaling Pathways in Human Bladder Cancer Cells, Pharmacognosy Magazine. (2020) 16, no. 71, 10.4103/pm.pm_21_20.

[111] Aggarwal B. B. , Tumour Necrosis Factors Receptor Associated Signalling Molecules and their Role in Activation of Apoptosis, JNK and NF-kappaB, Annals of the Rheumatic Diseases. (2000) 59, no. Suppl 1, i6–i16, 10.1136/ard.59.suppl_1.i6. 11053079 PMC1766635

[112] Seady M. , Schirmbeck G. , Taday J. et al., Curcumin Attenuates Neuroinflammatory Damage Induced by LPS: Implications for the Role of S100B, The Journal of Nutritional Biochemistry. (2025) 135, 10.1016/j.jnutbio.2024.109768. 39278425

[113] Iteire K. , Uwejigho R. , and Okonofua G. , Curcumin Attenuates Lipopolysaccharide-Induced Neuroinflammation and Memory Deficiency by Inhibiting Microglia Activation in Mice Hippocampus, Galician Med j. (2022) 29, no. 4, 10.21802/gmj.2022.4.5.

[114] Sharma N. , Sharma S. , and Nehru B. , Curcumin Protects Dopaminergic Neurons Against inflammation-mediated Damage and Improves Motor Dysfunction Induced by Single Intranigral Lipopolysaccharide Injection, Inflammopharmacology. (2017) 25, no. 3, 351–368, 10.1007/s10787-017-0346-z, 2-s2.0-85017465296. 28409389

[115] Khan M. S. , Muhammad T. , Ikram M. , and Kim M. O. , Dietary Supplementation of the Antioxidant Curcumin Halts Systemic LPS-Induced Neuroinflammation-Associated Neurodegeneration and Memory/Synaptic Impairment via the JNK/NF-*κ* B/Akt Signaling Pathway in Adult Rats, Oxidative Medicine and Cellular Longevity. (2019) 2019, 1–23, 10.1155/2019/7860650. PMC6885271 31827700

[116] Yıldız M. O. , Çelik H. , Caglayan C. et al., Neuromodulatory Effects of Hesperidin Against Sodium fluoride-induced Neurotoxicity in Rats: Involvement of Neuroinflammation, Endoplasmic Reticulum Stress, Apoptosis and Autophagy, NeuroToxicology. (2022) 90, 197–204, 10.1016/j.neuro.2022.04.002. 35413380

[117] Hajialyani M. , Hosein Farzaei M. , Echeverría J. , Nabavi S. M. , Uriarte E. , and Sobarzo-Sánchez E. , Hesperidin as a Neuroprotective Agent: A Review of Animal and Clinical Evidence, Molecules. (2019) 24, no. 3, 10.3390/molecules24030648, 2-s2.0-85061540322. PMC6384806 30759833

[118] Antunes M. S. , Ladd F. V. L. , Ladd A. A. B. L. , Moreira A. L. , Boeira S. P. , and Cattelan Souza L. , Hesperidin Protects Against Behavioral Alterations and Loss of Dopaminergic Neurons in 6-OHDA-lesioned Mice: The Role of Mitochondrial Dysfunction and Apoptosis, Metabolic Brain Disease. (2021) 36, no. 1, 153–167, 10.1007/s11011-020-00618-y. 33057922

[119] Bansal K. , Singh V. , Singh S. , and Mishra S. , Neuroprotective Potential of Hesperidin as Therapeutic Agent Inthe Treatment of Brain Disorders: Preclinical Evidence-basedReview, CMM. (2024) 24, no. 3, 316–326, 10.2174/1566524023666230320144722. 36959141

[120] Eciroglu-Sarban H. , Altin-Celik P. , Kelicen-Ugur P. , and Donmez-Altuntas H. , Neuroprotective Effects of Hesperidin and CK2 Inhibitor DRB on Aβ1-42-Induced Neurotoxicity in Differentiated SH-SY5Y Cells, Molecular Neurobiology. (2025) 62, no. 10, 12722–12735, 10.1007/s12035-025-05082-2. 40445481 PMC12433343

